# MIRF-Net: A Multimodal Data Fusion Framework for Intrapartum Fetal Risk Assessment

**DOI:** 10.3390/bioengineering13040385

**Published:** 2026-03-27

**Authors:** Yaosheng Lu, Yaqi Liang, Jieyun Bai, Ziduo Yang

**Affiliations:** Department of Electronic Engineering, College of Information Science and Technology, Jinan University, Guangzhou 510632, China; tluys@jnu.edu.cn (Y.L.); liangyaqi@stu2023.jnu.edu.cn (Y.L.); baijieyun@jnu.edu.cn (J.B.)

**Keywords:** intrapartum fetal risk assessment, multimodal fusion, cardiotocography (CTG), maternal metadata, PatchTST

## Abstract

Accurate assessment of hypoxia-related fetal risk during labour is essential for improving perinatal outcomes while avoiding unnecessary operative interventions. Although deep learning has shown promise for automated fetal risk assessment, most existing approaches rely on cardiotocography (CTG) alone; CTG interpretation is known to suffer from a high false-positive rate and may not fully reflect fetal status without complementary clinical context. To address this limitation, we propose MIRF-Net, a multimodal intrapartum fetal risk assessment framework that jointly models (i) CTG time-series signals, (ii) Gramian Angular Difference Field (GADF) images that encode global correlation structure of fetal heart rate, and (iii) structured maternal metadata. MIRF-Net combines a PatchTST encoder for CTG, a pretrained ResNet101 for GADF images, and an autoencoder for maternal metadata and then performs cross-modal interaction learning with a fusion Transformer for final risk prediction. Using 552 eligible CTG recordings from the public CTU-UHB intrapartum database, which were split into training, validation, and test sets at a ratio of 6:2:2, MIRF-Net outperforms representative baselines on the test set, achieving a quality index (QI) of 74.76%, AUC of 0.7413, and Brier score of 0.2537, indicating improved discrimination and better-calibrated risk probabilities. Ablation studies further confirm the complementary contributions of each modality and show that Transformer-based fusion yields the most consistent overall gains. These results suggest that MIRF-Net provides reliable decision support for intelligent intrapartum monitoring.

## 1. Introduction

Continuous assessment of fetal health during pregnancy is essential for ensuring maternal and neonatal safety throughout labor and delivery. The primary goal of intrapartum fetal monitoring is to identify potential hypoxia-related risk states in a timely manner before irreversible fetal injury occurs, thereby providing evidence for clinical intervention. Fetal heart rate (FHR) and uterine contraction (UC) signals are acquired from the maternal abdomen via ultrasound transducers to form cardiotocography (CTG), which is currently the primary clinical modality for intrapartum fetal monitoring [1]. The physiological patterns reflected in CTG signals provide critical information for intrapartum fetal risk assessment, and the timeliness and accuracy of these assessments directly affect the appropriateness of obstetric intervention decisions and the safety of maternal–neonatal outcomes. Abnormal CTG waveforms typically indicate fetal hypoxemia; failure to identify the associated risk in a timely manner may substantially increase the likelihood of long-term complications for both the mother and the fetus due to delayed intervention [2,3,4]. In the absence of clear evidence of significant fetal risk, unnecessary emergency operative deliveries should be avoided as much as possible—particularly in late labor—because the procedure itself entails considerable risks for both the mother and the fetus [5]. Therefore, developing reliable methods for intrapartum fetal risk assessment is of substantial clinical importance.

In routine clinical practice, fetal risk assessment still relies primarily on obstetricians’ comprehensive manual interpretation of CTG waveforms. This process is inevitably influenced by inter-physician differences in experience, workload, and subjective cognition, resulting in uncertainty and latency in identifying risk signals. Computerized CTG (cCTG) analysis systems are not affected by human factors such as fatigue or distraction, enabling around-the-clock operation and more systematic signal analysis to support clinical decision-making [6]. Existing cCTG approaches can be broadly categorized into machine learning (ML)-based and deep learning (DL)-based methods [7,8].

Early studies predominantly adopted ML approaches, manually extracting time-domain, frequency-domain, and time–frequency features from CTG or FHR signals and coupling them with classifiers to discriminate fetal status [9,10,11,12]. However, these methods largely depend on handcrafted feature engineering; feature definitions and selections are constrained by domain expertise and may introduce non-negligible measurement errors during secondary feature extraction [13,14]. Moreover, their performance is strongly influenced by sample size, signal quality in the database, and the intrinsic capability of the classifier itself [15,16], which limits the model’s ability to fully exploit complex dynamic information embedded in CTG signals.

To overcome the limitations imposed by reliance on handcrafted features, researchers have increasingly adopted DL models to perform end-to-end modeling of CTG signals, reducing the need for feature engineering while improving generalization. Existing studies have applied convolutional neural networks (CNNs), recurrent neural networks (RNNs), Transformers, and their variants to fetal status assessment [17,18,19,20,21,22,23]. In recent years, automated CTG interpretation has gradually shifted from single-signal modeling toward more clinically oriented deep learning frameworks, with greater emphasis on multi-source data integration, temporal dependency modeling, and broader validation [2,24,25]. Among these, multimodal fusion studies combining CTG with clinical information have shown that maternal and perinatal background variables can provide important complementary priors for risk assessment [24]. Meanwhile, Transformer- and patch-based models have also been introduced into CTG analysis to enhance the characterization of local patterns and long-range dependencies [26]. Recent large-scale and multicenter studies have further demonstrated the clinical application potential of deep learning for CTG interpretation, while indicating that issues such as class imbalance, model generalizability, and reliable discrimination still require further investigation [2,25,27]. However, most of these approaches focus on a single modality—primarily fetal heart rate (FHR)—while underutilizing uterine contraction (UC) signals and broader clinical context. This narrow focus limits their ability to capture the full complexity of intrapartum risk states and prevents effective exploitation of complementary multimodal information.

To address the above limitations, we propose MIRF-Net (Multimodal Intrapartum Risk Assessment Framework), a multimodal feature-fusion framework for intrapartum fetal risk assessment that jointly models CTG time-series signals, an image-based representation of FHR, and structured maternal clinical information within a unified model. Specifically, GAF-based image representations enhance characterization of complex heart-rate dynamics from a structural perspective, complementing patch-based temporal modeling [28], while maternal risk factors, e.g., hypertension, advanced maternal age, and nulliparity, provide clinically meaningful contextual priors that are difficult to infer from CTG alone [29,30]. The framework employs a CTG encoder, an image encoder, and a structured-data encoder to extract modality-specific features, and uses a Transformer-based multimodal fusion module to enable adaptive cross-modal interaction and joint modeling, thereby improving overall performance for intrapartum fetal hypoxia risk prediction.

In the intended clinical setting, MIRF-Net is positioned as a decision-support tool for intrapartum continuous monitoring rather than a fully autonomous diagnostic system. By jointly leveraging CTG time-series segments, GADF image representations generated from FHR signals, and structured maternal information, the model outputs the corresponding logits, predicted probabilities, and final normal/abnormal classification results, which can be used to prompt clinicians to further review potentially abnormal cases. Therefore, MIRF-Net is more appropriately used as an early-warning and second-opinion tool to support clinical risk screening, rather than to replace clinician judgment in final decision-making.

The main contributions of this work are as follows:

(1) We propose MIRF-Net, a multimodal intrapartum fetal risk assessment framework that jointly models the time-series signals of FHR and UC, the image-based representation of FHR, and maternal EHR information, enabling collaborative modeling of heterogeneous multi-source data and providing a more comprehensive and robust representation of complex intrapartum risk states.
(2) We introduce and apply PatchTST, a time-series Transformer architecture, to model FHR and UC signals. Through patch-based segmentation, instance normalization, and channel-independent processing, this approach effectively captures local dynamic variations and long-range temporal dependencies in CTG signals.
(3) We adopt a GAF-based image representation to transform one-dimensional FHR signals into two-dimensional images, complementing the modeling of complex heart-rate dynamics from a spatial-correlation perspective and enhancing representation of nonstationary signal characteristics.
(4) We employ a Transformer-based multimodal feature-fusion module to perform cross-modal interaction and joint representation learning in a unified feature space for intrapartum fetal hypoxia risk prediction.

The remainder of this paper is organized as follows: Section 2 describes the dataset and methodological details and presents MIRF-Net in full; Section 3 reports the experimental results; Section 4 discusses the results; and Section 5 concludes the paper and outlines future work.

## 2. Material and Methods

### 2.1. Data Source

**Figure 1:**
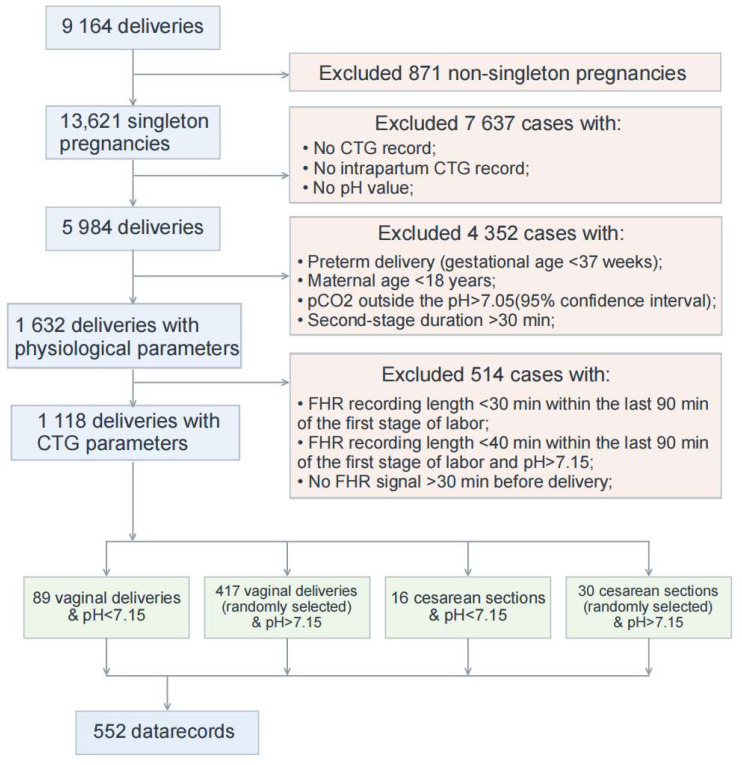
Screening process of the public CTU-UHB dataset.

In this study, fetal status assessment was based on 552 CTG recordings from the public CTU-UHB dataset. Among the 9164 raw intrapartum CTG recordings, 552 eligible recordings were finally included in this study according to the screening criteria adopted in previous studies on this database [31]. These criteria were established by integrating the inclusion and exclusion standards used in multiple previous CTG studies, and the corresponding sample selection workflow for the CTU-UHB database is illustrated in Figure 1. According to this workflow, the recordings not included in this study were mainly excluded for the following reasons: non-singleton pregnancies, missing CTG recordings, missing intrapartum CTG segments, missing pH values, failure to meet the relevant clinical eligibility criteria, or insufficient fetal heart rate (FHR) signal length within the target analysis window.

The original data were collected at the University Hospital Brno (Brno, Czech Republic) between April 2010 and August 2012 using STAN S21 and S31 electronic fetal monitoring devices (Neoventa Medical, Mölndal, Sweden) [31,32,33,34]. The raw CTG recordings are available from PhysioNet (https://www.physionet.org/content/ctu-uhb-ctgdb/1.0.0/, accessed on 20 March 2026). In addition, the CTU-UHB database provides summary maternal EHR information and delivery outcome-related data. The CTG signals were sampled at 4 Hz; all recordings were from singleton pregnancies with gestational age greater than 36 weeks. Each retained 30 min CTG segment had a signal loss rate of less than 50% [32].

As a high-quality dataset with expert annotations, CTU-UHB was labeled by nine senior obstetricians from six hospitals in the Czech Republic according to the FIGO guidelines, with a mean clinical experience of 15 years [13]. In this study, the last 30 min of CTG data before the end of the first stage of labor were used to build the classification model. Among the 552 included recordings, 330 were abnormal and 222 were normal. For dataset partitioning, stratified random splitting was performed according to fetal-status labels at a ratio of 6:2:2 to preserve class proportions across the training, validation, and test sets. Specifically, the training set contained 331 samples (198 abnormal and 133 normal), the validation set contained 110 samples (66 abnormal and 44 normal), and the test set contained 111 samples (66 abnormal and 45 normal).

For the structured maternal metadata used in this study (Age, Gravidity, Parity, and Diabetes), only Gravidity contained missing values (4/552, 0.72%), whereas the other three variables were complete. Missing metadata values were imputed using the mean value before standardization. The identification and reconstruction of missing or invalid signal points in FHR and UC are described in Section 2.2.

### 2.2. Data Preprocessing

Preprocessing raw biosignals is necessary to correct inaccuracies arising from real-world measurements [35]. For data selection, we used the last 30 min CTG segments of the first stage of labor from the CTU-UHB dataset. Each 30 min segment contains 7200 samples at a sampling rate of 4 Hz. Following prior studies [36,37], our preprocessing pipeline consists of three main steps: outlier detection, linear interpolation, and signal smoothing.

First, outlier detection is performed to identify and remove invalid data points; next, missing values are reconstructed via linear interpolation between valid samples; finally, smoothing is applied to further improve signal quality. Specifically, following the method of Xiao et al. [20], invalid points in FHR are defined as (1) values outside the physiologically plausible range (50–220 bpm) and (2) abrupt changes exceeding 25 bpm between two adjacent samples. For UC, invalid points are identified using the Pauta criterion (i.e., the 3σ rule) [38]. Concretely, a sliding-window strategy (window size = 500 in this study) is applied to compute the local mean and standard deviation within each window; any sample that deviates from the local mean by more than three standard deviations is labeled as invalid. In this study, these invalid points were treated as missing values and reconstructed by linear interpolation, followed by signal smoothing to improve signal quality.

**Figure 2:**
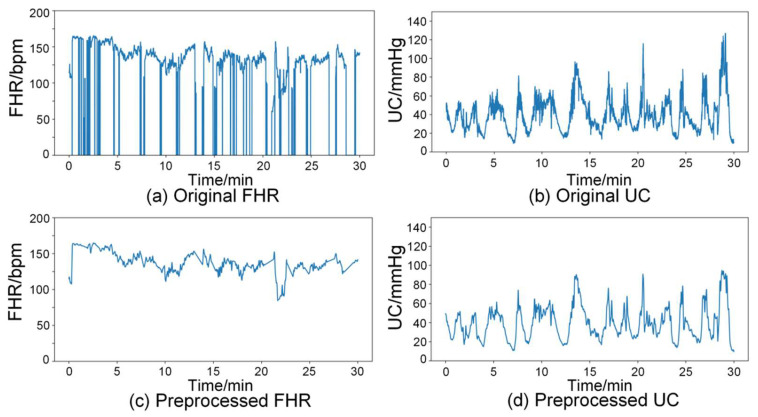
Comparison of FHR and UC signals before and after preprocessing: (**a**) raw FHR signal; (**b**) raw UC signal; (**c**) preprocessed FHR signal; (**d**) preprocessed UC signal. Abbreviations: FHR, fetal heart rate; UC, uterine contraction; bpm, beats per minute; mmHg, millimeters of mercury.

Figure 2 illustrates the preprocessing results for FHR and UC signals. Specifically, the FHR signal before and after preprocessing is shown in Figure 2a and Figure 2c, respectively, whereas the UC signal before and after preprocessing is shown in Figure 2b and Figure 2d, respectively.

### 2.3. Network Architecture

**Figure 3:**
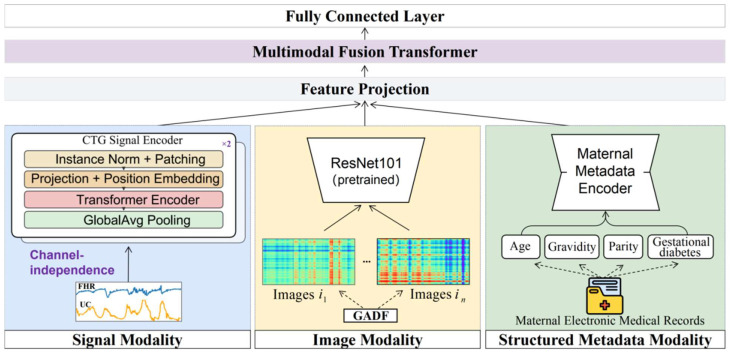
MIRF-Net for multimodal intrapartum fetal risk assessment: CTG time-series signals (FHR and UC), GADF images generated from FHR, and structured maternal metadata are encoded by modality-specific encoders to obtain embeddings; the three feature streams are linearly projected to a common dimensionality and organized into a sequence of modality tokens that is fed into a multimodal fusion Transformer to model cross-modal interactions; the fused representation is then passed to a fully connected classifier to output logits and predicted probabilities for normal vs. abnormal status.

As shown in Figure 3, the proposed MIRF-Net follows an overall paradigm of “modality-specific representation learning + cross-modal joint fusion” to integrate heterogeneous multi-source information in the intrapartum setting for comprehensive assessment of hypoxia-related fetal risk. The framework consists of three modality-specific encoders and a Transformer-based multimodal fusion module and produces risk predictions via a fully connected classification head. The input modalities include: (i) CTG time-series signals (FHR and UC); (ii) GADF images generated from FHR; and (iii) structured maternal metadata (age, gravidity, parity, and gestational diabetes). In the CTG branch, a PatchTST-based time-series Transformer performs multi-scale modeling of FHR and UC, leveraging patch partitioning and self-attention to capture both local morphological variations and long-range dependencies, yielding temporal embeddings. In the image branch, FHR is transformed via GADF into a two-dimensional image to explicitly encode global correlation structure, and a pretrained ResNet101 is used to extract texture/structural features, complementing the limitations of one-dimensional temporal representations in capturing complex nonlinear structures. In the metadata branch, a metadata autoencoder (MAE) is introduced for nonlinear compression and representation learning over structured features, producing compact latent variables that preserve key clinical priors while suppressing redundancy and noise. During fusion, embeddings from the three branches are first linearly projected to a common dimensionality and fed as a token sequence into the multimodal fusion Transformer, which models cross-modal interactions via multi-head self-attention to learn complementary information among temporal dynamics, image texture/structure, and maternal priors, producing a fused global representation. Finally, the fused representation is passed to a fully connected classifier for binary fetal status prediction, outputting the corresponding logits and predicted probabilities (e.g., normal vs. abnormal).

#### 2.3.1. CTG Signal Encoder Based on PatchTST

**Figure 4:**
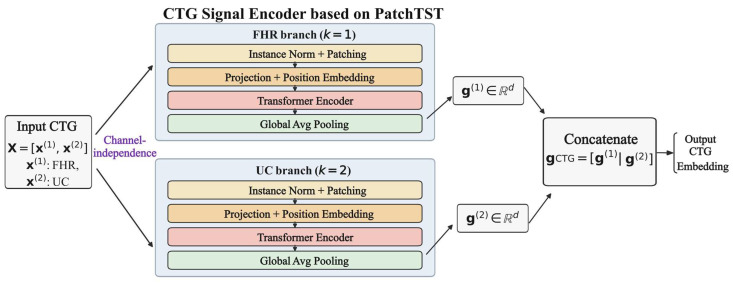
Architecture of the PatchTST-based CTG signal encoder. The input CTG sequence consists of two channels, FHR and UC. The model first applies instance normalization to each channel and then partitions the normalized sequence into multiple patches. Each patch is linearly projected and combined with positional embeddings before being fed into the Transformer encoder for feature modeling. Finally, global average pooling produces channel-level representations, which are concatenated to form the final CTG embedding.

The core of intrapartum fetal risk assessment lies in accurately modeling the complex temporal patterns in CTG signals, such as FHR and UC. CTG signals typically exhibit pronounced nonstationarity, multi-scale characteristics, and substantial inter-individual variability; their dynamics not only reflect the fetus’s current physiological state but are also closely associated with sustained intrauterine stress during labor. Therefore, a key challenge in CTG analysis is how to capture local dynamic variations while effectively modeling dependencies over long time horizons. To address this, we introduce PatchTST [23] as the CTG encoder to learn efficient and robust temporal representations of FHR and UC signals. The overall pipeline of the PatchTST-based CTG signal encoder is illustrated in Figure 4. The encoder adopts a channel-independent strategy to model FHR and UC separately while sharing the Transformer encoder parameters to enhance generalization.

Let a single CTG sample contain K channels (K = 2 in this study, corresponding to FHR and UC), and denote the one-dimensional time series of each channel as

(1) $$x^{\left(k\right)}=\left[x_{1}^{\left(k\right)},x_{2}^{\left(k\right)},\ldots ,x_{L}^{\left(k\right)}\right]\in {\mathbb{R}}^{L},\;k=1,\ldots ,K$$

where L denotes the length of the time series. Considering the substantial inter-subject differences in baseline level and amplitude distribution of CTG signals, we first apply instance normalization to each channel independently:

(2) $${\tilde{x}}^{\left(k\right)}=\frac{x^{\left(k\right)}-\mu^{\left(k\right)}}{\sigma^{\left(k\right)}}$$

Here, $\mu^{\left(k\right)}$ and $\sigma^{\left(k\right)}$ denote the mean and standard deviation of the time series in the k-th channel, respectively. This normalization is performed independently within each sample and each channel, helping to mitigate the effects of inter-subject variability, device differences, and baseline drift on model training.

Subsequently, PatchTST adopts a channel-independent modeling strategy, treating each channel in the multichannel CTG signal as an independent one-dimensional time series and modeling them separately. In the CTG setting, this design is physiologically well motivated, as FHR and UC originate from different mechanisms and thus exhibit markedly different statistical properties and dynamic patterns. Modeling FHR and UC separately can avoid inappropriate cross-channel attention interference, enabling more accurate learning of their respective temporal dynamics. Notably, although the channels are processed independently during the forward pass, they share the same Transformer encoder parameters, improving generalization while controlling model complexity.

After normalization and channel-wise separation, the one-dimensional time series of each channel is partitioned into patches. Given a patch length P and stride S, the i-th patch is defined as

(3) $$p_{i}^{\left(k\right)}=\left[{\tilde{x}}_{(i-1)S+1}^{\left(k\right)},{\tilde{x}}_{(i-1)S+2}^{\left(k\right)},\ldots ,{\tilde{x}}_{(i-1)S+P}^{\left(k\right)}\right]\in {\mathbb{R}}^{P}.$$

By applying zero padding at the end of the sequence to ensure full coverage, each channel can ultimately be represented as a patch sequence consisting of N patches, where $N=\left\lfloor \frac{L-P}{S}\right\rfloor +1$.

With the above patching operation, the number of input tokens to the Transformer is reduced from the original sequence length LLL to NNN, thereby lowering the self-attention complexity from $O(L^{2})$ to $O(N^{2})$ and substantially improving computational efficiency for long-duration CTG modeling.

Next, each patch is linearly embedded by projecting it into a d-dimensional feature space:

(4) $$z_{i}^{\left(k\right)}=W_{e}p_{i}^{\left(k\right)}+b_{e},\;z_{i}^{\left(k\right)}\in {\mathbb{R}}^{d},$$

where $W_{e}\in {\mathbb{R}}^{d\times P}$ and $b_{e}\in {\mathbb{R}}^{d}$ are learnable parameters. To preserve the relative positional information of patches in the original time series, a learnable positional embedding $e_{i}\in {\mathbb{R}}^{d}$ is introduced, and the resulting token representation fed into the Transformer is

(5) $$h_{i}^{\left(k\right)}=z_{i}^{\left(k\right)}+e_{i}.$$

The embedded patch sequence is fed into the Transformer encoder, where stacked multi-head self-attention and feed-forward networks jointly model local dynamic variations and long-range dependencies in CTG signals. The computation of a single self-attention layer is given by

(6) $$\mathrm{Attention}(Q,K,V)=\mathrm{softmax}\left(\frac{QK^{⊤}}{\sqrt{d}}\right)V,$$

Here, Q, K, and V denote the query, key, and value matrices obtained via linear projections of the input features, and $\sqrt{d}$ is used to scale the dot product to stabilize training.

After the output of the final Transformer encoder layer, global average pooling is applied over the patch dimension to obtain an overall temporal representation for each channel:

(7) $$g^{\left(k\right)}=\frac{1}{N}\sum_{i=1}^{N}h_{i}^{\left(k\right)}.$$

Finally, the feature representations of all channels are concatenated to form the overall representation of the CTG modality:

(8) $$g_{\mathrm{CTG}}=\left[g^{\left(1\right)}|g^{\left(2\right)}\right].$$

Notably, the original PatchTST is primarily designed for time-series forecasting, whereas in this work it is used as a temporal feature encoder for CTG signals. Accordingly, we discard its prediction head and instead use the high-level latent representations produced by the Transformer encoder as CTG features. These features are then fed, together with image-based FHR features and structured maternal clinical information, into the subsequent multimodal fusion module for comprehensive assessment of intrapartum fetal hypoxia risk.

#### 2.3.2. GADF Transformation and Image Encoder

In intrapartum fetal risk assessment, morphological variations in FHR waveforms often contain important pathological information, such as baseline drift, accelerations, decelerations, and variability. Traditional methods typically model one-dimensional time series directly; however, this representation cannot explicitly capture global correlation structures among temporally sampled points and may therefore overlook latent nonlinear dynamics in FHR signals. To further enhance the model’s capacity to represent complex temporal–morphological patterns, we introduce the GAF method [28] to map one-dimensional FHR time series into two-dimensional images and employ deep convolutional networks to extract their spatial structural features, thereby forming an image modality that complements CTG temporal features.

GAF is a widely used time-series-to-image transformation technique and has achieved strong performance in various time-series analysis tasks. A GAF image is generated by mapping a one-dimensional time series and can explicitly encode correlations among temporal samples in a two-dimensional space. Specifically, by applying the arccos function to the normalized FHR values, each time point in the sequence can be mapped into a polar coordinate system, yielding a new representation. The resulting Gramian matrix characterizes angular relationships between different time points, enabling the GAF image to preserve temporal ordering while capturing spatial structural patterns among samples.

Let a single FHR sequence be a time series of length L:

(9) $$x=[x_{1},x_{2},\ldots ,x_{L}]R^{L}.$$

First, to ensure the validity of the polar-coordinate mapping, the sequence is normalized to the interval [a,b] (with −1 ≤ a < b ≤ 1):

(10) $${\tilde{x}}_{i}=a+(b-a)\cdot \frac{x_{i}-\min(x)}{\max(x)-\min(x)},\;i=1,2,\ldots ,L.$$

This normalization ensures that the sequence values fall within [−1, 1], facilitating the subsequent arccos mapping and improving the information resolution of the GAF image. The normalized sequence is then mapped into polar coordinates, where the angle corresponding to each sample is defined as

(11) $$\phi_{i}=\arccos({\tilde{x}}_{i}),\;i=1,2,\ldots ,L.$$

After the polar-coordinate mapping, GAF converts the time series into a two-dimensional image by constructing a Gramian matrix. In this work, we use the Gramian Angular Difference Field (GADF) variant, which maps the angular difference between any two time points to a matrix element [39], defined as

(12) $$G_{i,j}=\cos(\phi_{i}-\phi_{j}),\;i,j=1,2,\ldots ,L,$$

where $G\in {\mathbb{R}}^{L\times L}$ denotes the GADF image matrix. This matrix explicitly captures the angular-difference relationships between temporal samples, thereby encoding the global structural information of the time-series signal in a two-dimensional space.

Because the directly generated GADF matrix has a size of L×L, long sequences would substantially increase the computational cost of the subsequent convolutional network. To standardize the input scale and improve computational efficiency, we resize it to a fixed resolution using bilinear interpolation:

(13) $$I^{GADF}=Resize(G,224,224),$$

Here, $I^{GADF}\in R^{224\times 224}$ denotes the final GADF image used as the model input, and i,j are pixel indices in the GADF image (corresponding to the resampled time indices).

**Figure 5:**
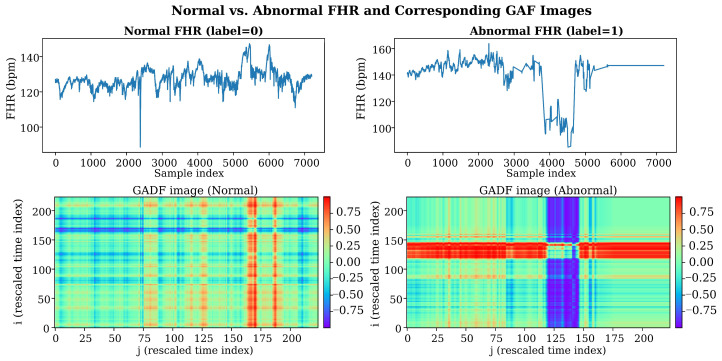
Examples of normal and abnormal FHR segments and their corresponding GADF images. The top row shows the FHR waveforms (bpm), and the bottom row shows the GADF images generated from the same FHR segments; the left column corresponds to a normal sample (label = 0) and the right column to an abnormal sample (label = 1). The horizontal axis j and vertical axis i of the GADF image are pixel indices (corresponding to resampled time indices); the pixel value $I_{i,j}$ represents the angular-difference relationship $\cos(\phi_{i}-\phi_{j})$ between two time points; and the main diagonal i=j indicates the self-pairing at the same time point.

Figure 5 presents an example of a normal (label = 0) and an abnormal (label = 1) FHR segment, together with their corresponding GADF images. Each pixel $I_{i,j}$ in the GADF image characterizes the angular-difference relationship $\cos(\phi_{i}-\phi_{j})$ between two time points (with indices i and j), thereby explicitly encoding global temporal correlation structure in a two-dimensional space; the main diagonal i = j corresponds to the self-pairing at the same time point. As shown in Figure 5, during intervals with pronounced decelerations or abrupt baseline shifts, abnormal samples tend to exhibit stronger structured texture variations in the GADF images (e.g., high-contrast bands or block-like regions), whereas normal samples display smoother texture distributions with weaker and more homogeneous structural differences. These observations suggest that the GADF representation can convert temporal–morphological variations in one-dimensional waveforms into two-dimensional spatial patterns that are easier for convolutional networks to capture, providing complementary information for subsequent image encoding and multimodal fusion.

After generating GADF images from the FHR signals, a pretrained ResNet101 network is employed as the image encoder to extract hierarchical visual features. During training, all backbone parameters are fully fine-tuned to allow the network to better adapt to the FHR-derived GADF image domain. The selection of ResNet101 is motivated by several considerations. First, the GADF representation mainly captures structural and texture-like patterns rather than natural-image semantic categories, making convolutional neural networks more suitable than transformer-based architectures under limited data conditions. Second, compared with shallower CNN backbones such as ResNet50, ResNet101 provides stronger hierarchical feature representation while maintaining stable optimization through residual learning. Third, pretrained CNN models provide robust initialization when the available training data are limited. Therefore, ResNet101 offers a suitable balance between representation capacity and training stability for modeling FHR-derived GADF images.

Let the output of ResNet101 with the final classification layer removed be a feature vector; the image encoding process can then be formulated as

(14) $$f_{img}=ResNet101(I^{GADF}),$$

where $f_{\mathrm{img}}\in {\mathbb{R}}^{d_{\mathrm{img}}}$ denotes the image modality embedding. By stacking residual blocks, ResNet101 can effectively extract texture and structural patterns from GADF images, thereby capturing discriminative information of FHR signals in the spatial correlation domain.

Finally, the resulting image modality feature $f_{\mathrm{img}}$ is fed, together with the CTG temporal feature $g_{CTG}$ obtained in the previous section and the structured maternal clinical features, into the subsequent multimodal fusion module to enable cross-modal interaction and joint representation learning, thereby supporting comprehensive assessment of intrapartum fetal hypoxia risk.

#### 2.3.3. Maternal Metadata Encoder

**Figure 6:**
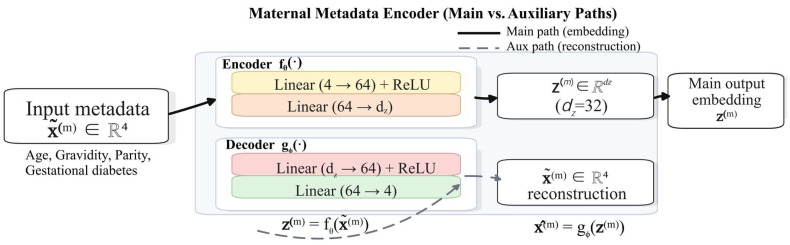
Schematic of the maternal structured-metadata encoder (MAE). Solid lines indicate the main embedding pathway used for multimodal fusion (${\tilde{x}}^{\left(m\right)}\to z^{\left(m\right)}$), whereas dashed lines denote the auxiliary reconstruction pathway during training ($z^{\left(m\right)}\to {\hat{x}}^{\left(m\right)}$).

In intrapartum fetal risk assessment, relying solely on CTG signals such as FHR and UC is often insufficient to capture risk heterogeneity arising from differences in maternal baseline status, obstetric history, and metabolic milieu. A clinical practice advisory on perinatal surveillance notes that a key goal of fetal monitoring is to identify potential risks of adverse fetal outcomes during high-risk pregnancy and labor, and maternal comorbidities (e.g., diabetes) are among the major indications for initiating monitoring and intensified risk assessment [40]. From a hypoxia-related pathophysiological perspective, when fetal oxygen supply decreases, compensatory mechanisms may partially sustain aerobic metabolism; however, once compensation is exhausted or oxygen reserves are insufficient, the fetus shifts to anaerobic metabolism, leading to adverse states such as acidemia. In pregnancies with disordered glucose metabolism, fetal hyperglycemia and hyperinsulinemia can increase fetal oxygen consumption and induce chronic intrauterine hypoxia, providing a mechanistic explanation for the link between the maternal metabolic environment and fetal susceptibility to hypoxia [41]. Therefore, we incorporate maternal electronic medical records (MEMRs) as a structured metadata modality into the multimodal framework to complement maternal background risk information that is difficult to represent using CTG alone; the architecture of the maternal structured-metadata encoder (MAE) is shown in Figure 6.

Combining data availability, clinical relevance, and perinatal risk evidence, this study selected maternal age, gravidity, parity, and gestational diabetes as the structured maternal features in MEMRs. Maternal age is statistically associated with the risk of hypoxia/asphyxia-related adverse outcomes; for example, Berglund et al. reported that maternal age ≥30 years is associated with increased risk of severe asphyxia in the context of asphyxia related to suboptimal intrapartum care [42]. Regarding more direct acidemia outcomes, a case–control study by Maisonneuve et al. on severe neonatal acidemia at term (e.g., umbilical arterial pH < 7.00) showed that maternal age ≥35 years at delivery is an independent risk factor [43].

For gravidity, although the mechanism linking it to acidemia remains unclear, Ogik et al. found a significant association between maternal gravidity and elevated umbilical arterial lactate and reported that primigravidas were more likely to have high umbilical lactate levels, providing evidence that differences in pregnancy history relate to hypoxia/metabolic-stress indicators [44]. With respect to parity, prior studies suggest systematic differences between nulliparous and multiparous women in labor progression and outcome risks. For instance, Cheng et al., in a large cohort of nulliparous women, analyzed the association between prolonged second stage of labor and adverse maternal and neonatal outcomes, indicating that nulliparas are more prone to labor prolongation and related risks, thereby supporting the inclusion of parity as an important obstetric-history covariate [45]. For gestational diabetes, Tarvonen et al. reported significant associations between gestational diabetes and abnormal intrapartum CTG patterns (e.g., late decelerations and ZigZag patterns) as well as hypoxia-related outcomes such as umbilical cord blood acidemia and low 5 min Apgar scores, suggesting an increased incidence of intrapartum fetal hypoxia in pregnancies complicated by gestational diabetes [46].

In summary, these four variables provide maternal risk priors from three complementary dimensions—epidemiological risk, obstetric history background, and metabolic environment—which complement CTG-based characterization of the fetus’s immediate responses and thereby enhance comprehensive assessment of intrapartum fetal risk states. In addition, given the limited sample size in the current study and the constraints on the availability and completeness of candidate maternal variables in the public dataset, we prioritized maternal features with stronger clinical interpretability and relatively more reliable data quality in order to reduce the influence of highly sparse or noisy variables on model stability.

At the data level, we represent the structured maternal features of each sample as a fixed-dimensional vector and align them one-to-one with the corresponding CTG segment using the record identifier. Let the metadata input be

(15) $$x^{\left(m\right)}=\left[\begin{array}{c} x_{\mathrm{age}}\\ x_{\mathrm{grav}}\\ x_{\mathrm{par}}\\ x_{\mathrm{dia}} \end{array}\right]\in {\mathbb{R}}^{4},$$

where $x_{age}$ denotes age, $x_{\mathrm{grav}}$ denotes gravidity, $x_{\mathrm{par}}$ denotes parity, and $x_{\mathrm{dia}}\in \{0,1\}$ is a binary indicator variable for gestational diabetes. Because the metadata variables differ substantially in scale and distribution, we apply z-score normalization to the numerical variables (age, gravidity, and parity) using statistics computed from the training set to improve training stability. For any numerical feature x, the standardized form is given by

(16) $$x\prime =\frac{x-\mu }{\sigma },$$

where μ and σ are the mean and standard deviation computed on the training set, respectively. Applying this standardization to $\left\{x_{age},x_{grav},x_{par}\right\}$ yields the preprocessed metadata vector:

(17) $${\tilde{x}}^{\left(m\right)}=\left[\begin{array}{c} {x^{\prime }}_{\mathrm{age}}\\ {x^{\prime }}_{\mathrm{grav}}\\ {x^{\prime }}_{\mathrm{par}}\\ x_{\mathrm{dia}} \end{array}\right]\in {\mathbb{R}}^{4}.$$

Although the metadata are low-dimensional, they are heterogeneous in type and may contain noise and redundant correlations; moreover, directly using raw features in small-sample settings can cause the model to either ignore this modality or overfit to it. To obtain more robust and fusion-ready metadata representations, we design a Metadata Autoencoder (MAE) to nonlinearly compress ${\tilde{x}}^{\left(m\right)}$ and learn a compact latent representation. As illustrated in Figure 6, the MAE consists of an encoder–decoder architecture: the input vector ${\tilde{x}}^{\left(m\right)}$ is mapped by the encoder $f_{\theta }(\cdot )$ to a latent variable $z^{\left(m\right)}$, which serves as the main output of the metadata branch for subsequent fusion with CTG/image modalities; meanwhile, to enforce representational fidelity during joint training and alleviate the risk that the metadata modality is ignored or suffers representation collapse during fusion, an auxiliary reconstruction pathway implemented by a decoder $g_{\phi }(\cdot )$ is introduced to produce a reconstruction ${\hat{x}}^{\left(m\right)}$.

Formally, the encoder maps the input to a low-dimensional latent variable:

(18) $$z^{\left(m\right)}=f_{\theta }\left({\tilde{x}}^{\left(m\right)}\right)\in {\mathbb{R}}^{d_{z}},$$

where $d_{z}$ denotes the latent dimensionality (set to $d_{z}$ = 32 in this study). The decoder generates a reconstruction of the input from the latent variable:

(19) $${\hat{x}}^{\left(m\right)}=g_{\phi }\left(z^{\left(m\right)}\right)\in {\mathbb{R}}^{4}.$$

In implementation, both the encoder and decoder are realized as lightweight multi-layer perceptrons (MLPs). The encoder consists of two fully connected layers with ReLU activations, and its forward mapping can be written as

(20) $$h^{\left(m\right)}=\mathrm{ReLU}\left(W_{1}{\tilde{x}}^{\left(m\right)}+b_{1}\right),$$

(21) $$z^{\left(m\right)}=W_{2}h^{\left(m\right)}+b_{2},$$

where $W_{1}\in {\mathbb{R}}^{64\times 4}$, $b_{1}\in {\mathbb{R}}^{64}$, $W_{2}\in {\mathbb{R}}^{d_{z}\times 64}\;$, and $b_{2}\in {\mathbb{R}}^{d_{z}}$ are learnable parameters. The decoder adopts a symmetric structure, and the reconstruction process is expressed as

(22) $${\hat{h}}^{\left(m\right)}=\mathrm{ReLU}\left(W_{3}z^{\left(m\right)}+b_{3}\right),$$

(23) $${\hat{x}}^{\left(m\right)}=W_{4}{\hat{h}}^{\left(m\right)}+b_{4},$$

where $W_{3}\in {\mathbb{R}}^{64\times d_{z}}$, $b_{3}\in {\mathbb{R}}^{64}$, $W_{4}\in {\mathbb{R}}^{4\times 64}$, and $b_{4}\in {\mathbb{R}}^{4}$ are learnable parameters. The reconstruction branch is used during joint training to regularize z\mathbf{z}z to preserve fidelity to maternal structured information, preventing the metadata modality from being ignored or suffering representation degradation in multimodal fusion; during inference, only the latent variable $z^{\left(m\right)}$ is used for fusion, whereas the reconstruction ${\hat{x}}^{\left(m\right)}$ is used solely as a training-time auxiliary regularizer and does not contribute to the final prediction. Through this nonlinear compression, the latent variable $z^{\left(m\right)}$ retains maternal risk-relevant information while suppressing redundant correlations, yielding a more robust structured representation that is better suited for subsequent multimodal modeling and fusion.

#### 2.3.4. Multimodal Fusion Transformer

In intrapartum fetal risk assessment, CTG time-series signals primarily characterize the fetus’s immediate responses and dynamic changes to intrauterine stimuli, whereas the GADF-based image modality explicitly represents the global correlation structure of the FHR sequence, and structured maternal clinical information provides background risk priors such as baseline status, obstetric history, and metabolic environment. The three modalities are markedly heterogeneous in information type and statistical characteristics: CTG features are dynamic representations combining local patterns with long-range dependencies, GADF image features emphasize spatial textures and structural patterns, and maternal metadata are low-dimensional, heterogeneous, and static variables with long-term associations with risk. Therefore, effectively aligning and modeling interactions among the three modalities within a unified framework is crucial for improving comprehensive assessment performance. Given the substantial differences across modalities in information source, statistical properties, and representation space, simple concatenation or fixed-weight summation often fails to explicitly capture adaptive complementarity—i.e., that a modality may be more critical for certain samples—and may cause weaker modalities to be overshadowed by dominant ones during training. To this end, we introduce a Transformer-encoder-based multimodal fusion module that learns cross-modal interaction dependencies via multi-head self-attention, thereby producing a more discriminative joint representation.

The above encoders produce three feature representations: the CTG branch output $g_{CTG}\in R^{d_{ctg}}$, the image branch output $f_{img}\in R^{d_{img}}$, and the metadata branch latent variable $z^{\left(m\right)}\in R^{d_{z}}$. Because the modalities differ in feature dimensionality and statistical distribution, we first apply three independent linear projections to map them into a shared space of dimension d, yielding three modality tokens.

(24) $$e^{\left(s\right)}=W_{s}g_{\mathrm{CTG}}+b_{s},e^{\left(i\right)}=W_{i}f_{\mathrm{img}}+b_{i},e^{\left(m\right)}=W_{m}z^{\left(m\right)}+b_{m},$$

where $e^{\left(s\right)}$,$e^{\left(i\right)}$,$e^{\left(m\right)}\in R^{d}$,$W_{s}\in {\mathbb{R}}^{d\times d_{\mathrm{ctg}}}$,$W_{i}\in {\mathbb{R}}^{d\times d_{\mathrm{img}}}$,$W_{m}\in {\mathbb{R}}^{d\times d_{z}}$ and the corresponding biases $b_{s}$,$b_{i}$,$b_{m}$ are learnable parameters. The three tokens are then stacked in a fixed order to form an input sequence of length 3:.

(25) $$X_{0}=\left[\begin{array}{c} e^{\left(s\right)}\\ e^{\left(i\right)}\\ e^{\left(m\right)} \end{array}\right]\in {\mathbb{R}}^{3\times d}.$$

To preserve token order and distinguish modality types, we add a learnable positional/modality embedding $P\in {\mathbb{R}}^{3\times d}$ to $X_{0}$, yielding the final input:

(26) $$H_{0}=X_{0}+P.$$

The multimodal fusion Transformer consists of L standard Transformer encoder blocks; each block contains multi-head self-attention (MHSA) and a feed-forward network (FFN and employs residual connections and LayerNorm to stabilize training. The computation in the l-th layer can be written as

(27) $${\tilde{H}}_{l}=\mathrm{LN}\left(H_{l-1}+\mathrm{MHSA}\left(H_{l-1}\right)\right),$$

(28) $$H_{l}=\mathrm{LN}\left({\tilde{H}}_{l}+\mathrm{FFN}\left({\tilde{H}}_{l}\right)\right),\;l=1,2,\ldots ,L,$$

where $H_{l}\in R^{3\times d}$ denotes the output of the l-th layer. The core of MHSA is the self-attention mechanism, whose single-head form is

(29) $$\mathrm{Attn}(Q,K,V)=\mathrm{softmax}\left(\frac{QK^{⊤}}{\sqrt{d_{h}}}\right)V,$$

where $Q=HW^{Q}$, $K=HW^{K}$, $V=HW^{V}$ and $d_{h}$ denote the per-head dimensions. Multi-head attention is implemented by computing multiple attention heads in parallel, concatenating them, and applying a linear projection:

(30) $$\mathrm{MHSA}(H)=\mathrm{Concat}({\mathrm{head}}_{1},\ldots ,{\mathrm{head}}_{h})W^{O}.$$

Through the above self-attention computation, the model can explicitly learn interdependencies among the three tokens (CTG, image, and metadata), e.g., relying more on CTG temporal variations for some samples while increasing the contribution of the image-texture or metadata token when pronounced decelerations occur or metabolic risk is high, thereby enabling adaptive cross-modal fusion.

After L fusion-encoding layers, we obtain $H_{L}\in R^{3\times d}$. We apply mean pooling over the token dimension to obtain a global fused representation:

(31) $$h_{\mathrm{fuse}}=\frac{1}{3}\sum_{t=1}^{3}H_{L}[t,:]\in {\mathbb{R}}^{d}.$$

The fetal risk probability is then produced by a fully connected classifier:

(32) $$\hat{y}=\sigma \left(w^{⊤}h_{\mathrm{fuse}}+b\right),$$

where σ(⋅) denotes the sigmoid function, and w and b are learnable parameters.

### 2.4. Loss Function

To jointly optimize intrapartum fetal risk classification performance and enforce fidelity of the structured maternal-metadata representation, we train the model end-to-end with a composite objective comprising a primary classification loss and an auxiliary reconstruction loss. Given a mini-batch $B={\left\{\left(x_{\mathrm{sig}}^{\left(m\right)},x_{\mathrm{img}}^{\left(m\right)},x_{\mathrm{ehr}}^{\left(m\right)},y^{\left(m\right)}\right)\right\}}_{m=1}^{M}$, $x_{\mathrm{sig}}$ denotes the CTG time-series input (FHR + UC), $x_{\mathrm{img}}^{\left(m\right)}$ denotes the GADF image generated from FHR, $x_{\mathrm{ehr}}\in {\mathbb{R}}^{4}$ denotes the structured maternal clinical features (age, gravidity, parity, and gestational diabetes), and y∈{1,…,C} is the class label (C=2 for binary classification).

The forward pass produces: (i) the classification-head logits $s^{\left(m\right)}\in {\mathbb{R}}^{C}$ and (ii) the reconstructed metadata vector ${\hat{x}}_{\mathrm{ehr}}^{\left(m\right)}\in {\mathbb{R}}^{4}$ from the metadata autoencoder. The overall training objective is defined as

(33) $$L=L_{\mathrm{cls}}+\lambda L_{\mathrm{rec}},$$

where $L_{\mathrm{cls}}$ is the primary classification loss, $L_{\mathrm{rec}}$ is the metadata reconstruction loss, and λ is a weighting coefficient (set to λ=0.5 in our implementation).

To mitigate over-confident predictions under small-sample and noisy-label settings, we use label-smoothing cross-entropy (LSCE) as the classification loss to improve generalization and training stability. For the m-th sample with ground-truth label $y^{\left(m\right)}$, the one-hot target is smoothed into a soft label ${\tilde{y}}^{\left(m\right)}\in {\mathbb{R}}^{C}$:

(34) $${\tilde{y}}_{c}^{\left(m\right)}=\left\{\begin{array}{ll} 1-\varepsilon , & c=y^{\left(m\right)},\\ \frac{\varepsilon }{C-1}, & c\neq y^{\left(m\right)} \end{array}\right.,$$

where ϵ∈[0,1] is the smoothing factor (ϵ=0.1 in our experiments).

Applying softmax to the logits $s^{\left(m\right)}$ yields the class-probability distribution:

(35) $$p_{c}^{\left(m\right)}=\mathrm{softmax}{\left(s^{\left(m\right)}\right)}_{c}=\frac{\exp\left(s_{c}^{\left(m\right)}\right)}{\sum_{k=1}^{C}\exp\left(s_{k}^{\left(m\right)}\right)}.$$

The LSCE is then defined as

(36) $$L_{\mathrm{cls}}=-\frac{1}{M}\sum_{m=1}^{M}\sum_{c=1}^{C}{\tilde{y}}_{c}^{\left(m\right)}\log p_{c}^{\left(m\right)}.$$

Label smoothing assigns a small probability mass to non-ground-truth classes, suppressing overly peaked predictive distributions, thereby mitigating overfitting and improving robustness to out-of-distribution or noisy samples.

Although the structured maternal features are low-dimensional, they are highly heterogeneous and can be overshadowed by dominant modalities (CTG time-series/images) during multimodal fusion. To preserve the metadata branch’s clinical-prior representation and prevent representation degradation, we introduce a reconstruction constraint during training; let the reconstruction output be ${\hat{x}}_{\mathrm{ehr}}^{\left(m\right)}$, and the reconstruction loss is defined as the mean squared error (MSE):

(37) $$L_{\mathrm{rec}}=\frac{1}{M}\sum_{m=1}^{M}{\left\|{\hat{x}}_{\mathrm{ehr}}^{\left(m\right)}-x_{\mathrm{ehr}}^{\left(m\right)}\right\|}_{2}^{2}.$$

This loss encourages the compressed metadata representation to retain key clinical information, thereby suppressing representation collapse of the metadata modality during joint training and improving its utility in the fusion attention mechanism.

During training, model parameters are updated by minimizing the joint loss L: gradients from $L_{\mathrm{cls}}$ update all three encoders, the fusion module, and the classification head, whereas gradients from $L_{\mathrm{rec}}$ primarily act on the metadata encoder/decoder branch to provide additional structured regularization. During inference, only the classification pathway is retained: the model outputs logits and predicted probabilities for risk discrimination, while the metadata reconstruction ${\hat{x}}_{\mathrm{ehr}}$ is discarded and used only as a training-time auxiliary regularizer.

### 2.5. Performance Evaluation

This study evaluates performance using eight metrics: ACC, SEN, SPE, QI, F1-score, AUC, MCC, and BS. For binary classification, abnormal CTG segments are treated as the positive class and normal segments as the negative class, yielding a confusion matrix with TN, FN, TP, and FP. ACC measures overall correctness; SEN and SPE quantify the correct identification of positive and negative samples, respectively; and QI (the geometric mean of SEN and SPE) provides a balanced summary that is less sensitive to class imbalance than ACC. To further account for imbalance, we report F1-score (harmonic mean of precision and recall) and MCC, which incorporates all four confusion matrix terms and ranges from −1 (complete disagreement) to 1 (perfect prediction). Discriminative ability is assessed by AUC, defined as the area under the ROC curve obtained by sweeping the decision threshold and plotting TPR versus FPR.

(38) $$ACC=\frac{TP+TN}{TP+FN+TN+FP},$$

(39) $$SEN=\frac{TP}{TP+FN},$$

(40) $$SPE=\frac{TN}{TN+FP},$$

(41) $$QI=\sqrt{SEN\cdot SPE},$$

(42) $$F1=\frac{2TP}{2TP+FP+FN},$$

(43) $$MCC=\frac{TP\times TN-FP\times FN}{\sqrt{\left(TP+FP)(TP+FN)(TN+FP)(TN+FN\right)}},$$

Finally, BS evaluates probabilistic accuracy/calibration as the mean squared error between predicted probabilities and observed outcomes, with lower values indicating better risk estimation.

(44) $$BS=\frac{1}{N}\sum_{i=1}^{N}{\left(p_{i}-o_{i}\right)}^{2},$$

where N denotes the total number of predicted categories, $p_{i}$ denotes the probability that a given prediction i occurs, and $o_{i}$ indicates whether the predicted event occurs. The value $o_{i}$ is 1 if the event occurs and 0 if it does not.

### 2.6. Implementation Details

The model was implemented and trained in the AutoDL cloud environment using Python 3.8 (Ubuntu 20.04), PyTorch 2.0.0, and CUDA 11.8; training was performed on a single vGPU with 48 GB of memory. We used the Adam optimizer with an initial learning rate of 1 × 10^−3^, weight decay of 1 × 10^−4^, a batch size of 64, and a maximum of 100 training epochs. Adam was chosen for its strong adaptivity and optimization efficiency across deep learning tasks; the learning rate of 1 × 10^−3^ is a commonly used default that typically balances convergence speed and training stability; and the weight decay of 1 × 10^−4^ helps prevent unbounded parameter growth and mitigates overfitting to some extent. The final model was selected as the checkpoint achieving the best performance on the validation set.

To improve the robustness and reproducibility of the experimental results, the CTU-UHB dataset was stratified according to fetal-status labels and split into training, validation, and test sets at a ratio of 6:2:2. To reduce the randomness associated with a single split, three independent experiments were conducted using three different random seeds (0, 42, and 3407). Specifically, each random seed corresponded to one independent data split, model training, and evaluation run, while all other experimental settings were kept unchanged. The final results are reported as mean ± standard deviation over the three runs.

## 3. Results

### 3.1. Comparison with Previous Models

To evaluate the effectiveness of MIRF-Net for intrapartum fetal risk assessment, we compare it with several representative baseline methods under a unified experimental setting. Specifically, all methods in Table 1 are evaluated on the same 552-record CTU-UHB cohort used in this study, with the same data-selection procedure, stratified train/validation/test splitting protocol, repeated-run setting, and evaluation metrics. As summarized in Table 1, the baselines include CNN–RNN hybrids (CNN-BiLSTM and CNN-RNN), convolution-based models (CTGNet, LARA, and 1D-SEResNet50), a Transformer variant (Medformer), and a multimodal method that integrates EHR information (MMDLA). Overall, MIRF-Net achieves the best or near-best performance on most metrics, indicating that jointly modeling CTG time-series signals (FHR + UC), GADF-based image representations, and structured maternal metadata provides complementary information and enables more robust risk discrimination.

Specifically, MIRF-Net attains an ACC of 74.63% (±6.05) and a QI of 74.76% (±6.40), suggesting a more balanced trade-off between sensitivity and specificity. It also achieves the highest F1-score (75.03% ± 6.20) and AUC (0.7413 ± 0.0663) among the compared methods and exhibits the strongest overall correlation with MCC = 0.4740 (±0.1058). In addition, MIRF-Net yields the lowest Brier score (BS = 0.2537 ± 0.0605), indicating improved probabilistic calibration relative to the baselines (Table 1).

Compared with the strongest single-modality CTG baseline, MIRF-Net still demonstrates consistent advantages. For example, the CNN-BiLSTM model of Xiao et al. (2022) [20] achieves ACC = 70.28% and AUC = 0.6800, whereas MIRF-Net improves these to 74.63% and 0.7413, respectively. LARA (Lin et al., 2024) [47] achieves extremely high sensitivity (SEN = 98.12%) but very low specificity (SPE = 28.23%), resulting in poor balance (QI = 52.56%); in contrast, MIRF-Net maintains a more symmetric trade-off (SEN = 74.86%, SPE = 74.72%), yielding a substantially higher QI. A similar pattern is observed for other CTG-only models (CTGNet, CNN-RNN, Medformer, and 1D-SEResNet50): MIRF-Net delivers more comprehensive overall performance rather than favoring one class.

Notably, although MMDLA (Cao et al., 2023) [24] integrates EHR information, its performance is limited by an extreme imbalance between sensitivity and specificity (e.g., SEN = 19.24% vs. SPE = 87.85%), leading to a low QI of 41.09%. By contrast, MIRF-Net employs a dedicated metadata encoder and a Transformer-based multimodal fusion strategy, enabling more robust and balanced risk assessment. Collectively, these results validate the effectiveness of MIRF-Net’s tri-modal design and fusion strategy for intrapartum fetal risk assessment.

**Figure 7:**
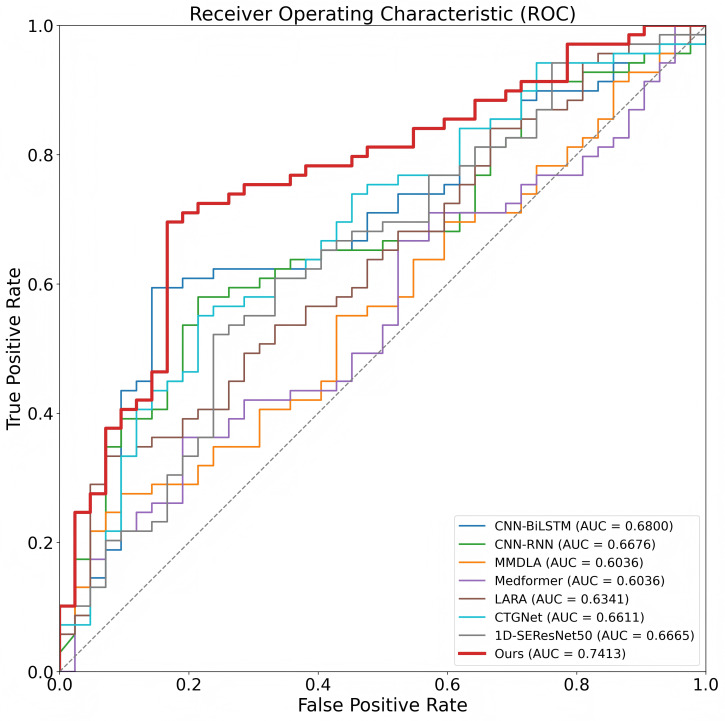
ROC curves of different methods on the CTU-UHB dataset. The dashed diagonal line indicates the performance of a random classifier (AUC = 0.5).

To more intuitively assess discriminative performance, Figure 7 shows the ROC curves of different methods on the test set. MIRF-Net (red curve) lies above the other baselines across most threshold ranges and is overall closer to the upper-left corner, with an AUC of 0.7413 that is markedly higher than the competing methods. This indicates that our model achieves a better true positive–false positive trade-off across decision thresholds, demonstrating more stable discrimination and stronger generalization robustness, which supports its potential for downstream clinical decision support.

### 3.2. Ablation Study on Modalities

**Figure 8:**
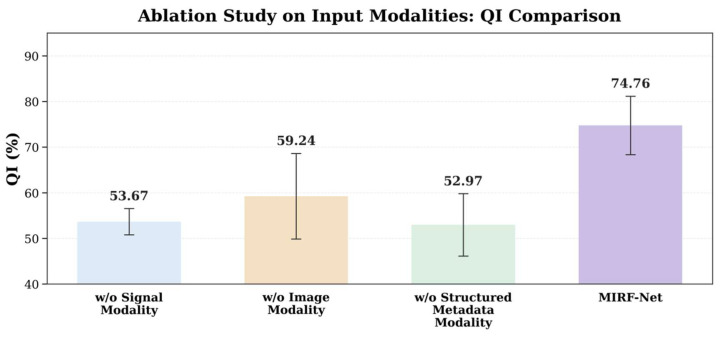
Modality ablation of MIRF-Net: QI (%) comparison.

To quantify the contribution of each modality in MIRF-Net and evaluate the effectiveness of fusion, we conducted modality ablation experiments under the same data split, training strategy, and evaluation pipeline: starting from the full tri-modal model (CTG time series + GADF images + structured metadata), we removed the CTG branch, the GADF-image branch, or the metadata branch to obtain three settings, namely w/o signal modality, w/o image modality, and w/o structured metadata modality. The test-set results are reported in Table 2, and the QI (%) comparison is shown in Figure 8.

Overall, the tri-modal model achieves the best and most balanced performance (ACC = 74.63%, QI = 74.76%, F1 = 75.03%, AUC = 0.7413, MCC = 0.4740) and the lowest BS = 0.2537 (Table 2), indicating that the three modalities are complementary and that fusion improves not only classification performance but also the reliability and calibration of probabilistic outputs.

Removing CTG time-series signals (w/o signal modality) causes a substantial performance drop, particularly in recognizing positive (abnormal CTG) cases: SEN decreases from 74.86% to 36.67%, leading to a QI reduction from 74.76% to 53.67% (−21.09 points), an AUC decrease from 0.7413 to 0.5818 (−0.1595), an MCC decrease from 0.4740 to 0.1680 (−0.3060), and a BS increase from 0.2537 to 0.3581 (+0.1044). Notably, SPE remains relatively high (78.64%) in this setting, suggesting a more conservative bias toward the negative class; this further indicates that CTG time-series signals are the core information source for intrapartum risk discrimination and are essential for stably capturing hypoxia-related dynamics.

Removing the GADF image modality (w/o image modality) also degrades performance but more moderately: QI drops from 74.76% to 59.24% (−15.52 points), AUC from 0.7413 to 0.6141 (−0.1272), MCC from 0.4740 to 0.1777 (−0.2963), and BS increases from 0.2537 to 0.3919 (+0.1382). This suggests that while the image branch is not the sole decisive information source, its 2D global structure representation complements pure time-series modeling of complex nonlinear morphological patterns and helps improve overall discrimination and stability.

Removing structured metadata (w/o structured metadata modality) results in one of the most pronounced declines, particularly in consistency-related metrics: QI decreases from 74.76% to 52.97% (−21.79 points, the largest among ablations), AUC from 0.7413 to 0.5642 (−0.1771, the largest), MCC from 0.4740 to 0.0561 (−0.4179, the largest), and BS increases from 0.2537 to 0.4562 (+0.2025, the largest). These findings highlight the non-negligible “prior” role of maternal structured clinical information (Age, Gravidity, Parity, and gestational diabetes): rather than improving a single metric, it provides baseline risk context and clinical priors that substantially enhance class-balanced performance (QI, MCC) and probability calibration (BS), thereby improving overall discriminative quality and robustness.

Taken together, the ablation results support two conclusions: (i) the CTG time-series modality is central to intrapartum risk assessment, and removing it markedly reduces recall for abnormal cases, and (ii) GADF images and maternal structured metadata provide complementary information, with the metadata modality being particularly important for improving overall metrics (QI, MCC) and calibration (BS). Tri-modal fusion maintains high sensitivity while preserving good specificity, yielding more stable, balanced, and trustworthy predictions in intrapartum fetal risk assessment.

### 3.3. Effect of Signal Composition

To examine how CTG composition affects MIRF-Net performance, we compared two input settings: using FHR only and using FHR + UC (Table 3). The results show consistent improvements across metrics after incorporating UC: with FHR only, ACC = 67.88%, QI = 68.48%, F1 = 68.45%, AUC = 0.7023, MCC = 0.3520, and BS = 0.3212; with FHR + UC, performance increases to ACC = 74.63%, QI = 74.76%, F1 = 75.03%, AUC = 0.7413, MCC = 0.4740, and BS = 0.2537. Specifically, QI, AUC, and MCC increase by 6.28 points, 0.0390, and 0.1220, respectively, while BS decreases by 0.0675, indicating that UC improves not only discriminative performance but also probabilistic calibration. Moreover, adding UC yields a more balanced identification of positive and negative classes, with SEN/SPE improving from 69.10%/68.75% to 74.86%/74.72%. This suggests that UC provides complementary cues reflecting the contraction–heart rate response, enabling more stable capture of hypoxia-related patterns and thereby improving overall performance.

### 3.4. Comparison of Fusion Strategies

**Figure 9:**
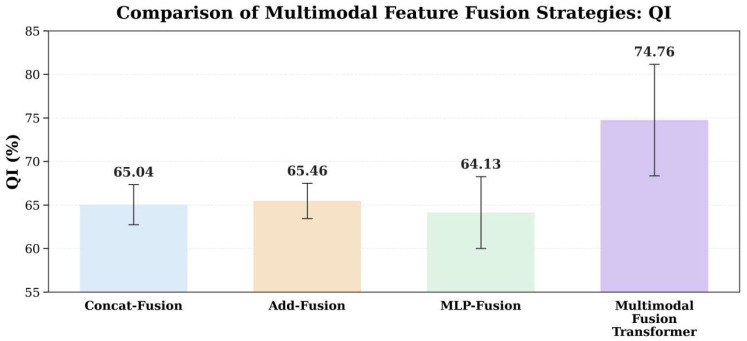
Comparison of multimodal feature fusion strategies in terms of QI (%).

To assess the role of the cross-modal fusion module, we compared four fusion strategies while keeping the three encoders and training settings unchanged: feature concatenation (Concat-Fusion), element-wise addition (Add-Fusion), MLP-based fusion (MLP-Fusion), and our Multimodal Fusion Transformer. Results under the same data split and evaluation protocol are reported in Table 4, and the QI comparison is shown in Figure 9.

The Multimodal Fusion Transformer achieves the best overall performance (ACC = 74.63%, QI = 74.76%, F1 = 75.03%, AUC = 0.7413, MCC = 0.4740) and the lowest BS (0.2537), indicating that the attention mechanism better exploits complementarity among CTG time series, GADF images, and structured maternal features, yielding more balanced discrimination and more reliable probability estimation. By contrast, although Concat-Fusion attains the highest SPE (80.44%), its SEN is markedly lower (52.71%), resulting in a QI of only 65.04%, which suggests a conservative decision boundary and an elevated risk of missed detections for high-risk (positive) cases. Add-Fusion performs slightly better than concatenation (QI = 65.46%), but it remains a linear scheme that cannot capture complex conditional dependencies across modalities and thus still lags substantially behind the Multimodal Fusion Transformer. As a nonlinear strategy, MLP-Fusion remains competitive in AUC (0.7172), yet it does not improve QI (64.13%) or MCC (0.2897), suggesting that with limited sample size and noisy labels, a pure MLP fusion may be more prone to unstable feature coupling or overfitting, thereby limiting generalization.

Overall, conventional fusion methods (Concat/Add/MLP) either provide only coarse integration or struggle to stably learn cross-modal complementarity, whereas the Multimodal Fusion Transformer uses multi-head self-attention to adaptively weight and interact cross-modal tokens, improving overall discrimination while markedly balancing SEN and SPE and achieving the largest gain in overall quality as reflected by QI. Therefore, we adopt the Multimodal Fusion Transformer as the default fusion module in MIRF-Net.

### 3.5. Contribution of Maternal Metadata

To systematically assess the added value of structured maternal metadata for intrapartum fetal hypoxia risk assessment, we kept the CTG time-series branch and the GADF-image branch of MIRF-Net fixed, varied only the input variable combination of the metadata branch (MAE), and compared performance under the same data split and evaluation protocol (Table 5 and Table 6) to quantify its marginal contribution as a third-modality prior.

**Figure 10:**
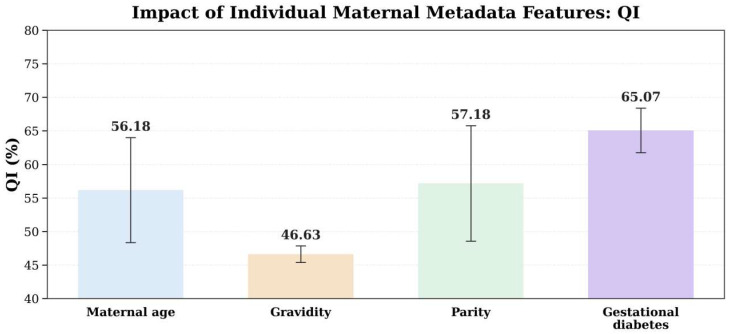
Marginal contribution of maternal structured metadata: QI (%) comparison under single-feature inputs.

The single-variable results (Table 5; QI in Figure 10) reveal marked heterogeneity across metadata variables: gestational diabetes alone yields the most stable and largest gains (higher QI, F1, AUC, and MCC, with lower BS), indicating the strongest risk informativeness; gravidity alone performs worst and even produces negative MCC, suggesting limited separability; maternal age and parity provide moderate priors but are insufficient for reliable standalone discrimination.

**Figure 11:**
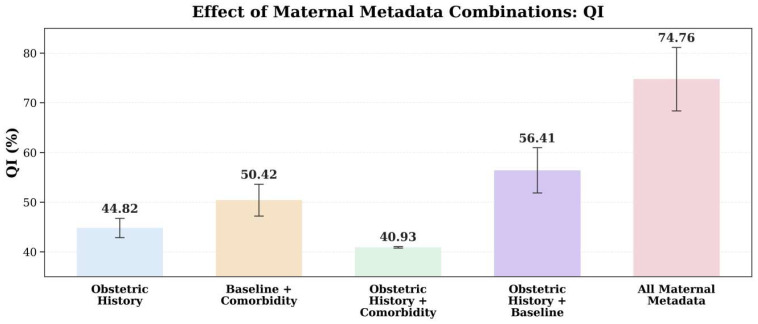
Marginal contribution of maternal structured metadata: QI (%) comparison under different metadata combinations.

The combination experiments (Table 6; QI in Figure 11) further show that the utility of metadata depends on how variable combinations shift error types: using “reproductive history” (gravidity + parity) yields high SPE but low SEN (69.32% vs. 29.03%), biasing predictions toward low risk and increasing missed detections; “age + gestational diabetes” yields higher SEN but lower SPE (72.22% vs. 35.42%), improving sensitivity at the cost of more false alarms; simply stacking reproductive-history and comorbidity information does not provide stable gains (QI = 40.93%, negative MCC), possibly introducing redundant or noisy priors; the relatively more balanced setting is “gravidity + parity + age” (SEN/SPE = 57.08%/55.91%, QI = 56.41%), but the improvement remains limited. Crucially, using the full set of metadata (gravidity + parity + age + gestational diabetes) achieves the best overall performance (concurrent improvements in QI, F1, AUC, and MCC with the lowest BS), indicating that a more comprehensive maternal prior enables individualized risk calibration during fusion while enhancing both discriminative power and probabilistic reliability.

### 3.6. Comparison of Image Encoder Architectures

Table 7 compares different image encoder backbones within the MIRF-Net framework, whereas Table 8 reports the corresponding trainable parameter statistics. Taken together, these results further reveal the trade-off between model complexity and predictive performance. Among the evaluated backbones, ResNet-101 achieved the best overall results, indicating that it provides the most suitable visual representation for FHR-derived GADF images in the present multimodal setting. Although the ResNet-101-based model contains 187.57 M trainable parameters, it consistently outperforms both the EfficientNet-based and Vision Transformer-based variants. By contrast, EfficientNet, despite having the smallest model size (124.90 M trainable parameters), yielded only moderate performance, suggesting that its efficiency-oriented design may be less effective in modeling the hierarchical structural patterns encoded by GADF representations. The Vision Transformer-based model, although having the largest number of trainable parameters (196.48 M), performed worst among the compared backbones, indicating that increasing parameter count alone does not necessarily improve performance under the current limited-cohort setting. Overall, these findings suggest that the effectiveness of the image encoder in MIRF-Net is not simply determined by model size; rather, ResNet-101 offers a more appropriate balance between representational capacity, optimization stability, and robustness.

### 3.7. Effect of ResNet Backbone Depth

Table 9 presents the effect of ResNet backbone depth on the performance of the image encoding branch. Overall, ResNet-101 achieved the best comprehensive performance among the evaluated ResNet variants. Compared with ResNet-50, ResNet-101 showed superior results across key metrics, including ACC, SEN, QI, F1, AUC, and MCC, indicating that a moderate increase in network depth helps capture the hierarchical structural features embedded in FHR-derived GADF images. However, when the network depth was further increased to ResNet-152, the model performance did not continue to improve. Although SPE increased, SEN, QI, AUC, and MCC all declined. This result suggests that, under the current small-cohort setting, simply increasing backbone depth does not necessarily lead to better overall discrimination performance, and may instead limit the model’s ability to achieve balanced recognition across classes. Therefore, ResNet-101 was selected as the final backbone for the image encoding branch.

### 3.8. Effect of Fine-Tuning Strategy on ResNet-101

Table 10 presents the comparison between full and partial fine-tuning strategies for the ResNet-101 image encoder. In the partial fine-tuning setting, the early layers of the backbone were frozen, while only higher-level residual blocks (layer3 and layer4) were updated during training. As shown in Table 10, full fine-tuning consistently yields superior performance across all evaluation metrics, including ACC, SEN, SPE, QI, F1, AUC, MCC, and BS. Compared with partial fine-tuning, full fine-tuning improves both classification effectiveness and probability calibration. This result suggests that freezing early layers restricts the ability of the pretrained backbone to adapt to the FHR-derived GADF image domain. Therefore, full fine-tuning was adopted as the final training strategy for the ResNet-101 image encoder.

### 3.9. Hyperparameter Sensitivity

#### 3.9.1. Effect of Patch Size and Stride on CTG Signal Encoding Performance

To analyze the hyperparameter sensitivity of PatchTST for CTG encoding, we conducted comparative experiments on patch length and stride while keeping the data split, training settings, and multimodal architecture unchanged, with results summarized in Table 11.

The results indicate that there is an optimal range of patch scales, where patch = 64 and stride = 32 achieves the best and most balanced performance (ACC = 74.63%, QI = 74.76%, F1 = 75.03%, AUC = 0.7413, MCC = 0.4740, BS = 0.2537). Excessive overlap markedly degrades performance; for example, with patch = 64 and stride = 16, QI drops to 49.92%, AUC to 0.5383, and MCC approaches 0, suggesting that redundant tokens may increase optimization difficulty and weaken attention focus on salient patterns. Increasing the patch size does not necessarily yield gains: although patch = 128 and stride = 32 remains relatively strong (QI = 71.46%, AUC = 0.7165), performance fluctuates more; patch = 128 and stride = 24 leads to a pronounced SEN/SPE imbalance, indicating that overly large patches may impair the resolution of short-term morphological changes. Overall, these findings highlight the need to balance local-detail resolution against modeling stability and redundancy control; accordingly, we use patch = 64 and stride = 32 as the default setting in subsequent experiments.

#### 3.9.2. Hyperparameter Sensitivity to the Number of Attention Heads in the Multimodal Fusion Transformer

To assess MIRF-Net’s sensitivity to the multi-head self-attention configuration during cross-modal fusion, we fixed the Fusion Transformer at two layers and varied only the number of heads (2/4/8/16), with all other settings unchanged (Table 12). The results show that the number of heads has a substantial impact on fusion performance, and four heads yields the best and most balanced overall results (ACC = 74.63%, QI = 74.76%, F1 = 75.03%, MCC = 0.4740, BS = 0.2537). Two heads leads to pronounced degradation due to limited cross-modal interactions (QI = 56.70%, MCC = 0.1401). Although eight heads achieves the highest AUC (0.7586), it does not yield corresponding gains in QI, MCC, or calibration; increasing to 16 heads further degrades performance (QI = 55.62%, BS = 0.4329), possibly due to training instability. Therefore, we adopt a two-layer, four-head configuration as the default setting in subsequent experiments.

#### 3.9.3. Hyperparameter Sensitivity to the Number of Layers in the Multimodal Fusion Transformer

To analyze the effect of fusion-module depth on cross-modal interaction modeling, we fixed the number of attention heads at 4 and kept all other settings unchanged, varying only the number of Fusion Transformer layers and comparing performance (Table 13). The results show that two layers achieves the best and most stable performance (ACC = 74.63%, QI = 74.76%, F1 = 75.03%, AUC = 0.7413, MCC = 0.4740, with the lowest BS of 0.2537), indicating that a shallow fusion stack is sufficient to capture key cross-modal complementarity while balancing discrimination and calibration. Increasing the depth to 3–4 layers leads to pronounced degradation (ACC ≈ 57%, QI = 47.90–57.93%, BS = 0.4243–0.4267), possibly due to redundant interactions, noise propagation, and training instability in the small-sample regime; although five layers shows a partial rebound (ACC = 69.77%, QI = 65.18%, AUC = 0.7418), it still underperforms the two-layer setting and yields a higher BS (0.3023). Therefore, we adopt a two-layer (Heads = 4) configuration as the default setting in subsequent experiments.

#### 3.9.4. Effect of the Label Smoothing Coefficient on Model Performance

To investigate the effect of the label smoothing coefficient on model performance, comparative experiments were conducted using different coefficients, as summarized in Table 14. The results show that the label smoothing coefficient has a notable impact on overall discriminative performance and class balance. When the coefficient was set to 0.1, the model achieved the best overall performance, with ACC, QI, F1, AUC, and MCC reaching 74.63%, 74.76%, 75.03%, 0.7413, and 0.4740, respectively, while BS was the lowest at 0.2537. When the coefficient increased to 0.2 and 0.3, performance declined markedly. In particular, at 0.2, SEN decreased substantially whereas SPE increased noticeably, indicating insufficient detection of abnormal samples. At 0.3, the metrics deteriorated further, suggesting that excessive label smoothing weakens the model’s ability to learn class boundaries. Therefore, 0.1 was adopted as the default setting in the subsequent experiments.

#### 3.9.5. Effect of the Reconstruction Loss Weight λ on Model Performance

To investigate the effect of the reconstruction loss weight λ on model performance, comparative experiments were conducted using different values of λ, as summarized in Table 15. Overall, λ has a notable influence on classification performance and training stability. When λ is small, the reconstruction branch provides limited auxiliary supervision, leading to relatively poor overall performance. For example, when λ = 0.1 and 0.3, ACC, QI, AUC, and MCC are all clearly lower than the best results. Although λ = 0.3 increases SEN to 78.19%, SPE is only 54.00%, indicating insufficient class balance. When λ = 0.5, the model achieves the best overall performance, with ACC, QI, F1, AUC, and MCC reaching 74.63%, 74.76%, 75.03%, 0.7413, and 0.4740, respectively, while BS is the lowest at 0.2537. When λ further increases to 0.7 and 1.0, the performance declines again and metric fluctuations become larger, suggesting that excessive reconstruction loss weakens optimization for the main classification task. Overall, the reconstruction loss weight λ has a clear impact on model performance. The results show that λ=0.5 provides the best balance in discriminative ability, class balance, and output stability; therefore, it was adopted as the default setting in the subsequent experiments.

## 4. Discussion

This study targets automated assessment of intrapartum fetal hypoxia-related risk by proposing a multimodal fusion framework, MIRF-Net, and validating its effectiveness under a unified data split and evaluation protocol. Compared with prior work that primarily relies on a single signal or a single representation pathway, MIRF-Net jointly models three complementary sources—CTG time-series signals (FHR + UC), a structured image-based representation of FHR (GADF), and structured maternal metadata (Age, Gravidity, Parity, Gestational diabetes)—and explicitly learns cross-modal interactions via a Multimodal Fusion Transformer. In terms of overall metrics, MIRF-Net yields more balanced improvements in QI, F1, MCC, and AUC while maintaining better probabilistic calibration as reflected by a lower BS, demonstrating a favorable balance between discrimination and stability. This is particularly important for intrapartum risk assessment, where decision thresholds often need to be adjusted according to clinical resources, risk preferences, and intervention costs; thus, practical utility depends more on the overall trade-off between false alarms and missed detections across thresholds than on maximizing sensitivity or specificity alone.

From a clinical translation perspective, the value of MIRF-Net does not lie in replacing clinician judgment, but in providing more stable abnormal-risk cues during intrapartum continuous monitoring. In particular, besides improving discriminative performance, the model also demonstrates better probability calibration, as reflected by the lower BS, suggesting that its outputs may be more suitable for clinical risk stratification and early warning. In practice, for cases classified as abnormal or with a relatively high predicted probability of abnormal status, the system may prompt clinicians to further review the original CTG tracing, maternal condition, and labor progression, thereby increasing vigilance for potentially high-risk cases. In this sense, MIRF-Net is better positioned as an early-warning and second-opinion tool to support clinical review and continuous monitoring, rather than as a system that directly replaces obstetricians in intervention decisions.

In comparisons with representative methods (Table 1), MIRF-Net shows a more consistent advantage in overall interpretive quality and comprehensive performance. Specifically, MIRF-Net achieves best or near-best ACC (74.63%), QI (74.76%), F1 (75.03%), and MCC (0.4740), attains a lower BS (0.2537), and maintains an AUC of 0.7413, indicating that under the current experimental setting, the model improves discrimination ability, class balance, and the stability of risk probabilities. However, from a clinical perspective, this AUC level should still be interpreted cautiously. Although it is competitive relative to the compared baselines and is accompanied by better probability calibration and more balanced classification performance, it is still insufficient to support fully autonomous clinical decision-making. A more appropriate interpretation is that the current results demonstrate the potential of MIRF-Net as an auxiliary risk-screening and decision-support tool, especially for abnormal-case flagging and second-opinion support in continuous monitoring scenarios. In contrast, some baselines exhibit pronounced imbalance; for instance, Lin et al.’s [47] LARA attains very high SEN (98.12%) but extremely low SPE (28.23%), constraining QI (52.56%) and potentially imposing a high false-positive burden in screening scenarios, while Xiao et al.’s [20] CNN-BiLSTM is comparatively strong among conventional deep models yet still lags behind MIRF-Net, particularly in overall balance and calibration (BS improving from 0.2972 to 0.2537). We attribute these differences to MIRF-Net’s multimodal design: PatchTST provides a more effective multiscale representation for CTG time series, the GADF–ResNet branch offers texture features sensitive to global correlation structure, and the metadata branch supplies individualized risk priors via a compact representation; their complementary synergy within the fusion Transformer better meets the combined needs of discrimination, class balance, and probabilistic usability in intrapartum risk assessment.

Modality ablations further clarify the contribution and synergy of each modality (Table 2). Removing the signal modality reduces SEN to 36.67% and markedly lowers QI, confirming that CTG time-series information remains the primary basis for risk discrimination. Meanwhile, the signal composition study shows that adding UC further improves overall performance (Table 3), highlighting the importance of contraction–heart rate coupling for characterizing hypoxia-related risk. For the image modality, removing the GADF branch decreases QI from 74.76% to 59.24% (Table 2), indicating that mapping 1D FHR into a 2D structure via GADF provides discriminative cues complementary to pure time-series modeling. This aligns with the method design: GADF explicitly encodes angular-difference relations between time points in 2D space, enabling convolutional networks to capture structure changes associated with abnormal morphology from texture patterns, thereby compensating for limitations of 1D representations in modeling complex nonlinear structure.

The maternal-metadata contribution is mainly reflected in improved overall decision quality and more stable risk probabilities (Table 4 and Table 5). When the structured metadata modality is removed, BS increases from 0.2537 to 0.4562 (Table 2), indicating substantially worse calibration; moreover, comparisons across metadata combinations (Table 5 and Table 6) show that settings including risk-relevant variables such as gestational diabetes and age exert a stronger impact on overall performance, suggesting that maternal context provides conditional priors for similar CTG morphologies and supports more individualized risk baselines during fusion. This finding is also consistent with the perinatal clinical consensus that maternal metabolic and obstetric background influences fetal tolerance to hypoxia.

In analyses of fusion strategies and hyperparameter sensitivity, we observed that compact yet effective cross-modal interactions are more conducive to generalization. Transformer-based fusion outperforms concatenation, addition, and MLP fusion (Table 4), supporting the advantage of explicit attention interactions for learning modality complementarity. Conversely, increasing the depth of the fusion Transformer does not yield sustained gains; performance drops markedly when layers increase from 2 to 3–4 (Table 10), suggesting that deeper fusion stacks may introduce overfitting or unstable cross-modal dependencies under the present data scale and noise level, while results on the number of heads likewise indicate that moderate configurations are more robust (Table 9). These observations emphasize that, in small-sample medical settings with highly heterogeneous modalities, fusion-module capacity should be matched to data scale, regularization, and modality noise levels.

Despite the favorable results in intrapartum hypoxia-related risk prediction, this study has several limitations. First, the public CTU-UHB dataset used here has a relatively limited sample size and primarily covers term singleton deliveries, without including more complex subgroups such as preterm births, multiple gestations, or advanced maternal age. It should be noted that 552 recordings still represent a relatively limited sample size for a deep multimodal architecture. Therefore, in the present study, we adopted a compact fusion design, modality-specific encoders, pretrained feature extraction, and repeated experimental runs to reduce the risk of overfitting and improve the robustness of the results. More importantly, the present study has not yet conducted external validation on an independent external cohort. For clinical AI systems, external validation is a critical step for assessing model robustness, transportability, and real-world applicability. Therefore, the generalizability of MIRF-Net across different hospitals, monitoring devices, patient populations, and clinical workflows still needs to be further validated in larger multicenter, cross-device, and prospective real-world cohorts. In addition, internal validation on a single dataset usually benefits from distributional consistency and may therefore overestimate model performance in real clinical environments. Second, while MIRF-Net’s multi-branch fusion architecture improves discriminative accuracy, it also increases memory footprint and inference latency. Compared with unimodal models, the higher hardware requirements may limit deployment efficiency on bedside devices or in real-time monitoring scenarios. Future work may reduce computational cost via lightweight backbones, knowledge distillation, pruning/quantization, and simplified fusion modules, and evaluate real-time performance and stability using online sliding-window inference.

## 5. Conclusions

This study addresses the need for automated assessment of intrapartum fetal hypoxia-related risk by proposing a multimodal data-fusion framework, MIRF-Net. To our knowledge, this framework is the first to jointly model CTG time-series signals, GADF-based spatial representations of fetal heart rate, and heterogeneous structured maternal information within a unified end-to-end architecture. Methodologically, MIRF-Net uses a PatchTST-based temporal Transformer to capture multi-scale dynamics and long-range dependencies in CTG, leverages a pretrained ResNet to learn representations of the global correlation structure in GADF images, and designs a metadata autoencoder (MAE) to robustly compress low-dimensional, heterogeneous MEMR features; it then employs a multimodal fusion Transformer to model cross-modal interactions for adaptive fusion and joint discrimination across information sources. On the CTU-UHB dataset, MIRF-Net achieves superior overall performance and more reliable probabilistic outputs compared with representative baselines. Ablation and comparative experiments further show that the CTG time-series modality provides the primary discriminative evidence, while GADF images and maternal metadata offer complementary information; incorporating UC and attention-based fusion further improves the sensitivity–specificity trade-off and calibration quality. Future work will focus on multicenter external validation and lightweight deployment to facilitate practical adoption in bedside and real-time monitoring settings.

## Figures and Tables

**Table 1:** Comparison with Representative Previous Models on Intrapartum Fetal Risk Assessment under a Unified CTU-UHB Evaluation Protocol.

Author	Method	Input Modality	ACC (%)	SEN (%)	SPE (%)	QI (%)	F1 (%)	AUC	MCC	BS
Xiao et al., (2022) [20]	CNN-BiLSTM	FHR + UC	70.28 (±2.84)	80.86 (±1.56)	64.08 (±5.12)	71.92 (±2.65)	70.79 (±3.02)	0.6800 (±0.0117)	0.4323 (±0.0384)	0.2972 (±0.0284)
Lin et al., (2024) [47]	LARA	FHR + UC	53.36 (±2.25)	**98.12** **(±2.01)**	28.23 (±2.44)	52.56 (±1.95)	49.61 (±2.40)	0.6341 (±0.0780)	0.3241 (±0.0184)	0.4664 (±0.0225)
Ogasawara et al., (2021) [30]	CTGNet	FHR + UC	66.09 (±3.93)	55.33 (±5.57)	72.13 (±3.07)	63.11 (±4.15)	66.22 (±4.11)	0.6611 (±0.0283)	0.2711 (±0.0737)	0.3391 (±0.0393)
Liang et al., (2022) [17]	CNN-RNN	FHR + UC	63.52 (±2.63)	71.65 (±6.88)	58.64 (±0.97)	64.73 (±3.20)	64.24 (±2.24)	0.6676 (±0.0222)	0.2924 (±0.0701)	0.3648 (±0.0263)
Wang et al., (2025) [48]	Medformer	FHR + UC	49.37 (±1.54)	58.73 (±6.07)	43.85 (±2.23)	50.61 (±1.81)	50.10 (±1.00)	0.5778 (±0.0218)	0.0261 (±0.0433)	0.5063 (±0.0154)
Park et al., (2025) [2]	1D-SEResNet50	FHR + UC	65.77 (±3.72)	59.22 (±6.31)	69.64 (±2.97)	64.15 (±4.21)	66.14 (±3.92)	0.6665 (±0.0539)	0.2805 (±0.0730)	0.3423 (±0.0372)
Cao et al., (2023) [24]	MMDLA	FHR + UC + MEMRs	63.17 (±3.65)	19.24 (±1.56)	**87.85** **(±2.63)**	41.09 (±2.16)	58.07 (±3.79)	0.6036 (±0.0690)	0.0977 (±0.0551)	0.3683 (±0.0365)
**Ours**	**MIRF-Net**	**FHR + UC** + **GADF Images + MEMRs**	**74.63** **(±6.05)**	74.86 (±7.39)	74.72 (±6.08)	**74.76** **(±6.40)**	**75.03** **(±6.20)**	**0.7413** **(±0.0663)**	**0.4740** **(±0.1058)**	**0.2537** **(±0.0605)**

Bold values indicate the best result for each metric among the compared methods.

**Table 2:** Ablation Study on Input Modalities for Intrapartum Fetal Risk Assessment.

Input Modality Setting	ACC (%)	SEN (%)	SPE (%)	QI (%)	F1 (%)	AUC	MCC	BS
w/o Signal Modality	64.19 (±7.02)	36.67 (±0.86)	**78.64** **(±6.65)**	53.67 (±2.87)	62.84 (±6.94)	0.5818 (±0.0175)	0.1680 (±0.0852)	0.3581 (±0.0702)
w/o Image Modality	60.81 (±8.80)	54.37 (±12.49)	64.81 (±6.19)	59.24 (±9.38)	61.38 (±8.76)	0.6141 (±0.0118)	0.1777 (±0.0179)	0.3919 (±0.0980)
w/o Structured Metadata Modality	54.38 (±6.70)	49.96 (±8.49)	57.50 (±6.94)	52.97 (±6.83)	55.29 (±7.78)	0.5642 (±0.0209)	0.0561 (±0.1319)	0.4562 (±0.0667)
MIRF-Net	**74.63** **(±6.05)**	**74.86** **(±7.39)**	74.72 (±6.08)	**74.76** **(±6.40)**	**75.03** **(±6.20)**	**0.7413** **(±0.0663)**	**0.4740** **(±0.1058)**	**0.2537** **(±0.0605)**

Bold values indicate the best result for each metric among the compared methods.

**Table 3:** Effect of CTG signal composition on MIRF-Net performance.

Input Signal	ACC (%)	SEN (%)	SPE (%)	QI (%)	F1 (%)	AUC	MCC	BS
FHR	67.88 (±6.57)	69.10 (±18.73)	68.75 (±3.67)	68.48 (±9.67)	68.45 (±6.68)	0.7023 (±0.0614)	0.3520 (±0.0129)	0.3212 (±0.0668)
**FHR** **+ UC**	**74.63** **(±6.05)**	**74.86** **(±7.39)**	**74.72** **(±6.08)**	**74.76** **(±6.40)**	**75.03** **(±6.20)**	**0.7413** **(±0.0663)**	**0.4740** **(±0.1058)**	**0.2537** **(±0.0605)**

Bold values indicate the best result for each metric among the compared methods.

**Table 4:** Comparison of Multimodal Feature Fusion Strategies.

Fusion Method	ACC (%)	SEN (%)	SPE (%)	QI (%)	F1 (%)	AUC	MCC	BS
Concat-Fusion	71.03 (±5.13)	52.71 (±2.24)	**80.44** **(±5.89)**	65.04 (±2.30)	70.60 (±5.00)	0.7267 (±0.0381)	0.3436 (±0.0631)	0.2897 (±0.0513)
Add-Fusion	68.43 (±2.79)	57.64 (±3.18)	74.47 (±3.51)	65.46 (±2.02)	68.66 (±3.16)	0.6607 (±0.0283)	0.3150 (±0.0338)	0.3157 (±0.0279)
MLP-Fusion	67.40 (±5.13)	55.83 (±4.17)	73.71 (±4.70)	64.13 (±4.13)	67.56 (±5.43)	0.7172 (±0.0619)	0.2897 (±0.0734)	0.3260 (±0.0513)
Multimodal Fusion Transformer	**74.63** **(±6.05)**	**74.86** **(±7.39)**	74.72 (±6.08)	**74.76** **(±6.40)**	**75.03** **(±6.20)**	**0.7413** **(±0.0663)**	**0.4740** **(±0.1058)**	**0.2537** **(±0.0605)**

Bold values indicate the best result for each metric among the compared methods.

**Table 5:** Impact of individual maternal metadata features on the multimodal model performance.

Maternal Metadata Features	ACC (%)	SEN (%)	SPE (%)	QI (%)	F1 (%)	AUC	MCC	BS
Maternal age	59.62 (±7.99)	47.64 (±9.10)	**66.44** **(±6.38)**	56.18 (±7.84)	59.95 (±8.47)	0.6151 (±0.0954)	0.1326 (±0.1385)	0.4038 (±0.0799)
Gravidity	48.54 (±0.11)	40.97 (±3.47)	53.26 (±1.74)	46.63 (±1.22)	49.01 (±0.06)	0.4805 (±0.0195)	−0.0566 (±0.0169)	0.5146 (±0.0011)
Parity	55.04 (±6.60)	65.62 (±12.78)	49.98 (±6.00)	57.18 (±8.60)	55.97 (±7.35)	0.5497 (±0.0838)	0.1396 (±0.1560)	0.4496 (±0.0660)
Gestational diabetes	**65.58** **(±4.87)**	**65.76** **(±2.59)**	64.77 (±8.06)	**65.07** **(±3.32)**	**66.22** **(±5.13)**	**0.6963** **(±0.0519)**	**0.2921** **(±0.0581)**	**0.3442** **(±0.0487)**

Bold values indicate the best result for each metric among the compared methods.

**Table 6:** Effect of different maternal metadata combinations on the multimodal model performance.

Group	Maternal Metadata Features	ACC (%)	SEN (%)	SPE (%)	QI (%)	F1 (%)	AUC	MCC	BS
Obstetric History	Gravidity + Parity	53.68 (±4.13)	29.03 (±0.14)	69.32 (±5.68)	44.82 (±1.95)	52.22 (±3.69)	0.5072 (±0.0355)	−0.0161 (±0.0618)	0.4632 (±0.0413)
Baseline + Comorbidity	Maternal Age + Gestational diabetes	47.78 (±2.22)	72.22 (±9.16)	35.42 (±2.73)	50.42 (±3.21)	47.60 (±2.32)	0.5086 (±0.0429)	0.0747 (±0.0866)	0.5222 (±0.0222)
Obstetric History + Comorbidity	Gravidity + Parity + Gestational diabetes	48.75 (±2.81)	26.94 (±1.94)	62.54 (±4.96)	40.93 (±0.15)	47.83 (±2.34)	0.4931 (±0.0319)	−0.1086 (±0.0291)	0.5125 (±0.0281)
Obstetric History + Baseline	Gravidity + Parity +Maternal Age	56.40 (±4.95)	57.08 (±3.66)	55.91 (±6.80)	56.41 (±4.55)	57.41 (±5.58)	0.5517 (±0.0589)	0.1202 (±0.0801)	0.4360 (±0.0495)
**All Maternal** **Metadata**	**Gravidity + Parity + Maternal Age + Gestational diabetes**	**74.63** **(±6.05)**	**74.86** **(±7.39)**	**74.72** **(±6.08)**	**74.76** **(±6.40)**	**75.03** **(±6.20)**	**0.7413** **(±0.0663)**	**0.4740** **(±0.1058)**	**0.2537** **(±0.0605)**

Bold values indicate the best result for each metric among the compared methods.

**Table 7:** Comparison of Different Image Encoder Backbones within the MIRF-Net Framework.

Image Encoder Backbone	ACC (%)	SEN (%)	SPE (%)	QI (%)	F1 (%)	AUC	MCC	BS
EfficientNet	58.65 (±2.37)	58.97 (±5.20)	58.39 (±3.02)	58.58 (±2.76)	59.65 (±3.14)	0.6000 (±0.0356)	0.1629 (±0.0437)	0.4135 (±0.0237)
Vision Transformer	53.66 (±3.79)	46.39 (±3.00)	57.46 (±5.08)	51.54 (±2.62)	54.62 (±4.53)	0.5470 (±0.0627)	0.0348 (±0.0484)	0.4634 (±0.0379)
**ResNet-101**	**74.63** **(±6.05)**	**74.86** **(±7.39)**	**74.72** **(±6.08)**	**74.76** **(±6.40)**	**75.03** **(±6.20)**	**0.7413** **(±0.0663)**	**0.4740** **(±0.1058)**	**0.2537** **(±0.0605)**

Bold values indicate the best result for each metric among the compared methods.

**Table 8:** Trainable Parameter Statistics of MIRF-Net under Different Image Encoder Backbones.

Image Encoder Backbone	Trainable Parameters
EfficientNet	124.90 M
Vision Transformer	196.48 M
ResNet-101	187.57 M

**Table 9:** Effect of ResNet Backbone Depth on Model Performance in the Image Encoding Branch.

ResNet Depth	ACC (%)	SEN (%)	SPE (%)	QI (%)	F1 (%)	AUC	MCC	BS
ResNet-50	57.16 (±2.21)	23.61 (±1.39)	78.64 (±1.36)	43.08 (±1.64)	53.88 (±2.40)	0.4937 (±0.0220)	0.0263 (±0.0322)	0.4284 (±0.0221)
**ResNet-101**	**74.63** **(±6.05)**	**74.86** **(±7.39)**	74.72 (±6.08)	**74.76** **(±6.40)**	**75.03** **(±6.20)**	**0.7413** **(±0.0663)**	**0.4740** **(±0.1058)**	**0.2537** **(±0.0605)**
ResNet-152	67.59 (±4.83)	37.43 (±2.53)	**86.66** **(±5.17)**	55.86 (±0.97)	65.78 (±4.83)	0.6362 (±0.0314)	0.2390 (±0.0451)	0.3241 (±0.0483)

Bold values indicate the best result for each metric among the compared methods.

**Table 10:** Comparison of Full and Partial Fine-Tuning Strategies for ResNet-101 in the Image Encoding Branch.

Fine-Tuning Strategies	ACC (%)	SEN (%)	SPE (%)	QI (%)	F1 (%)	AUC	MCC	BS
Partial fine-tuning	69.28 (±5.73)	67.08 (±5.32)	70.45 (±5.87)	68.73 (±5.41)	69.79 (±5.98)	0.6934 (±0.0990)	0.3583 (±0.0919)	0.3072 (±0.0573)
**Full fine-tuning**	**74.63** **(±6.05)**	**74.86** **(±7.39)**	**74.72** **(±6.08)**	**74.76** **(±6.40)**	**75.03** **(±6.20)**	**0.7413** **(±0.0663)**	**0.4740** **(±0.1058)**	**0.2537** **(±0.0605)**

Bold values indicate the best result for each metric among the compared methods.

**Table 11:** Hyperparameter Sensitivity of Patch Size and Stride in CTG Signal Encoding.

Patch Length	Stride	ACC (%)	SEN (%)	SPE (%)	QI (%)	F1 (%)	AUC	MCC	BS
64	16	51.52 (±5.26)	46.04 (±2.97)	54.20 (±6.06)	49.92 (±4.15)	52.61 (±5.94)	0.5383 (±0.0594)	−0.0004 (±0.0788)	0.4848 (±0.0526)
**64**	**32**	**74.63** **(±6.05)**	**74.86** **(±7.39)**	74.72 (±6.08)	**74.76** **(±6.40)**	**75.03** **(±6.20)**	**0.7413** **(±0.0663)**	**0.4740** **(±0.1058)**	**0.2537** **(±0.0605)**
96	24	58.97 (±8.10)	59.65 (±9.22)	58.75 (±8.47)	59.13 (±8.39)	59.81 (±8.57)	0.6523 (±0.1286)	0.1686 (±0.1462)	0.4103 (±0.0810)
128	24	51.95 (±6.39)	37.43 (±2.53)	59.13 (±8.09)	46.85 (±2.72)	52.47 (±6.72)	0.5686 (±0.0697)	−0.0350 (±0.0678)	0.4805 (±0.0639)
128	32	71.92 (±9.93)	68.89 (±18.66)	**74.75** **(±6.06)**	71.46 (±12.25)	72.16 (±10.16)	0.7165 (±0.1178)	0.4129 (±0.2097)	0.2808 (±0.0993)

Bold values indicate the best result for each metric among the compared methods.

**Table 12:** Effect of the number of attention heads in the multimodal fusion Transformer on model performance (with a fixed number of layers).

Layers	Heads	ACC (%)	SEN (%)	SPE (%)	QI (%)	F1 (%)	AUC	MCC	BS
2	2	59.70 (±5.33)	48.96 (±8.06)	65.95 (±3.46)	56.70 (±5.73)	60.16 (±6.04)	0.6327 (±0.0808)	0.1401 (±0.0923)	0.4030 (±0.0533)
**2**	**4**	**74.63** **(±6.05)**	**74.86** **(±7.39)**	74.72 (±6.08)	**74.76** **(±6.40)**	**75.03** **(±6.20)**	0.7413 (±0.0663)	**0.4740** **(±0.1058)**	**0.2537** **(±0.0605)**
2	8	71.93 (±2.49)	61.67 (±2.32)	**77.48** **(±2.14)**	69.11 (±1.79)	72.10 (±2.80)	**0.7586** **(±0.0200)**	0.3856 (±0.0317)	0.2807 (±0.0249)
2	16	56.71 (±2.66)	51.53 (±2.64)	60.04 (±2.46)	55.62 (±2.56)	57.11 (±2.75)	0.5387 (±0.0384)	0.1132 (±0.0494)	0.4329 (±0.0266)

Bold values indicate the best result for each metric among the compared methods.

**Table 13:** Effect of the number of layers in the multimodal fusion Transformer on model performance (with a fixed number of attention heads).

Layers	Heads	ACC (%)	SEN (%)	SPE (%)	QI (%)	F1 (%)	AUC	MCC	BS
**2**	**4**	**74.63** **(±6.05)**	**74.86** **(±7.39)**	74.72 (±6.08)	**74.76** **(±6.40)**	**75.03** **(±6.20)**	0.7413 (±0.0663)	**0.4740** **(±0.1058)**	**0.2537** **(±0.0605)**
3	4	57.57 (±8.50)	58.19 (±17.13)	58.30 (±5.70)	57.93 (±10.79)	58.44 (±9.08)	0.6155 (±0.1262)	0.1462 (±0.1902)	0.4243 (±0.0850)
4	4	57.33 (±5.62)	32.43 (±3.53)	70.83 (±4.17)	47.90 (±3.87)	56.58 (±6.25)	0.5473 (±0.0498)	0.0322 (±0.0747)	0.4267 (±0.0562)
5	4	69.77 (±5.33)	54.17 (±12.19)	**79.32** **(±1.96)**	65.18 (±7.14)	69.38 (±6.08)	**0.7418** **(±0.0625)**	0.3337 (±0.1013)	0.3023 (±0.0533)

Bold values indicate the best result for each metric among the compared methods.

**Table 14:** Hyperparameter Sensitivity of the Label Smoothing Coefficient.

Label Smoothing Coefficient	ACC (%)	SEN (%)	SPE (%)	QI (%)	F1 (%)	AUC	MCC	BS
**0.1**	**74.63** **(±6.05)**	**74.86** **(±7.39)**	74.72 (±6.08)	**74.76** **(±6.40)**	**75.03** **(±6.20)**	**0.7413** **(±0.0663)**	**0.4740** **(±0.1058)**	**0.2537** **(±0.0605)**
0.2	66.17 (±7.25)	42.78 (±4.46)	**78.77** **(±5.65)**	58.03 (±4.98)	65.24 (±7.47)	0.6911 (±0.0835)	0.2274 (±0.1030)	0.3383 (±0.0725)
0.3	55.71 (±4.33)	57.08 (±3.66)	54.83 (±5.03)	55.93 (±4.21)	56.78 (±5.10)	0.5556 (±0.0796)	0.1095 (±0.0719)	0.4429 (±0.0433)

Bold values indicate the best result for each metric among the compared methods.

**Table 15:** Hyperparameter Sensitivity of the Reconstruction Loss Weight λ.

Reconstruction Loss Weight λ	ACC (%)	SEN (%)	SPE (%)	QI (%)	F1 (%)	AUC	MCC	BS
0.1	64.05 (±2.17)	61.67 (±2.32)	65.32 (±2.06)	63.46 (±2.06)	64.88 (±2.61)	0.6640 (±0.0172)	0.2567 (±0.0373)	0.3595 (±0.0217)
0.3	62.33 (±3.44)	**78.19** **(±6.08)**	54.00 (±4.09)	64.97 (±4.86)	63.07 (±4.07)	0.6775 (±0.0259)	0.3033 (±0.0793)	0.3767 (±0.0344)
**0.5**	**74.63** **(±6.05)**	74.86 (±7.39)	**74.72** **(±6.08)**	**74.76** **(±6.40)**	**75.03** **(±6.20)**	**0.7413** **(±0.0663)**	**0.4740** **(±0.1058)**	**0.2537** **(±0.0605)**
0.7	71.74 (±7.50)	72.50 (±16.01)	72.42 (±4.17)	72.24 (±9.83)	72.19 (±7.78)	0.7400 (±0.1215)	0.4216 (±0.1633)	0.2826 (±0.0750)
1.0	59.06 (±7.75)	61.04 (±15.42)	58.92 (±5.98)	59.72 (±9.78)	59.92 (±8.31)	0.5607 (±0.0984)	0.1801 (±0.1712)	0.4094 (±0.0775)

Bold values indicate the best result for each metric among the compared methods.

## Data Availability

The data presented in this study are openly available in [PhysioNet (CTU-UHB Intrapartum Cardiotocography Database)] at [https://www.physionet.org/content/ctu-uhb-ctgdb/1.0.0/, accessed on 20 March 2026].
